# Characterization of genetic alterations in brain metastases from non‐small cell lung cancer

**DOI:** 10.1002/2211-5463.12501

**Published:** 2018-08-30

**Authors:** Li Liao, Xiaoyu Ji, Mengxi Ge, Qiong Zhan, Ruofan Huang, Xiaohua Liang, Xinli Zhou

**Affiliations:** ^1^ Department of Oncology Huashan Hospital Fudan University Shanghai China; ^2^ Department of Oncology Shanghai Medical College Fudan University Shanghai China

**Keywords:** brain metastases, *EGFR*, heterogeneity, non‐small cell lung cancer, *TP53*

## Abstract

Brain metastasis (BM) is the primary contributor to mortality in non‐small cell lung cancer (NSCLC) patients. Although the findings of NSCLC genetic sequencing studies suggest the potential for personalizing therapeutic approaches, the genetic profiles and underlying mechanisms of BM progression remain poorly understood. Here, we investigated the genetic profiles of brain metastases from NSCLC in six patients with primary tumors and corresponding BM samples via whole exome sequencing and targeted panel sequencing. We have demonstrated considerable genetic heterogeneity between primary lung cancer and corresponding brain metastases specimens. High‐frequency mutations were found in *NOTCH2*,*NOTCH2NL*,*FANCD2*,*EGFR*, and *TP53*. Additionally, *EGFR* and *TP53* consistently exhibited high frequencies of mutation between primary tumors and corresponding brain metastases. The implication is that most of the genetic alterations may be acquired or lost during malignant progression, and the stable *EGFR* and *TP53* mutational status between paired primary tumors and metastatic sites confirms that most mutations detected on analysis of the primary tumor or metastases are sufficient for clinical decision‐making, and suggest there is no need to re‐biopsy recurrent tumors or metastases for most NSCLC patients.

**Abbreviations**
BM: brain metastasis
BP: biological process
CC: cellular components
GO: gene ontology
KEGG: Kyoto Encyclopedia of Genes and Genomes
MF: molecular functions
NSCLC: non‐small cell lung cancer
SNV: somatic nucleotide variant
WES: whole exome sequencing

Lung cancer has become the most frequently occurring tumor and the leading cause of cancer‐related death 1. Eighty‐five per cent of lung cancers are classified as non‐small cell lung cancer (NSCLC). Adenocarcinoma cases account for the largest number of NSCLC patients 2. Median survival for patients with NSCLC is 13 months 3, and metastasis is the critical contributor to mortality rate. The brain is one of the most common organs for lung cancer metastasis, in which metastasis indicates poor prognosis. Approximately 10–25% of patients with lung cancer have brain metastases when the cancer is first diagnosed, and 40–50% of these eventually develop brain metastases during the course of their disease 4. The median survival of these patients decreases to 2 months 5.

Precision medicine and targeted therapies are defined as individual treatments based on an individual patient's mutant molecular profiles. Genetic sequencing studies of NSCLC have demonstrated complex subclones with genetic heterogeneity, both within primary tumors and between primary and lymphatic metastases 6, 7. Nevertheless, little is known regarding genetic heterogeneity of brain metastases in NSCLC. Furthermore, the heterogeneity of molecular profiles between primary tumors and brain metastases or heterogeneity that develops during the malignant process may limit the efficacy of individual treatments. Therefore, there is an urgent need to explore mutations in brain metastases and to determine individualized therapy regimens. This study focused on investigating the altered genes in brain metastases from NSCLC and explored potential targeted treatments for driver mutations.

We carried out whole exome sequencing (WES) and deep target panel sequencing on six primary tumors and their corresponding brain metastasis samples, respectively. Then, we systematically analyzed somatic nucleotide variants (SNVs) and predicted the possibly or probably pathogenic genes. We then performed bioinformatic analysis to explore potential brain metastasis (BM)‐associated driver genes or SNVs, as well as enriched biological processes (BP), molecular functions (MF) and signaling pathways.

## Materials and methods

### Clinical and sample characteristics

All protocols of the study were approved by the International Review Board of Fudan University Affiliated Huashan Hospital. Only BM from NSCLC was included in this study. We included six patients with a mean age of 53.5 years at diagnosis (range, 31–64 years) who underwent surgery or biopsy and had pathologically confirmed NSCL carcinoma and corresponding brain metastases. For three patients, brain metastases developed 14–24 months after primary tumor surgery. All primary and paired‐metastases tumor tissue specimens were subsequently investigated by next generation sequence whole exome analysis. Another three patients were diagnosed with lung cancer and brain metastases simultaneously. The experiments were undertaken with the understanding and written consent of each subject. The study methodologies conformed to the standards set by the Declaration of Helsinki. The details of clinical characteristics are displayed in Table 1.

**Table 1:** Clinical and histopathological characteristics. Interval: time between primary tumor and brain metastasis; tumor size: maximum diameter of the tumor; stage: classification of lymph nodes and distal organs metastasis status according to the international system for staging lung cancer; TNM: stage of initial diagnosis. CT, chemotherapy; ND, not determined; TNM, the first tumor, lymph node, metastasis classification

ID	P1	P2	P3	P4	P5	P6
Age (years)	58	33	57	56	67	64
Sex	Male	Male	Male	Female	Male	Male
Histological type	Adenocarcinoma	Adenocarcinoma	Adenocarcinoma	Adenocarcinoma	Adenocarcinoma	Adenocarcinoma
Interval	16 months	14 months	24 months	Synchronous	Synchronous	Synchronous
Tumor size (cm)						
Primary	4.4	2.5	5.5	4.0	3.9	7.0
Metastases	2.8	5.0	ND	ND	ND	ND
Smoker	No	No	No	No	No	No
Stage	IIA	IIIA	IIB	IV	IV	IV
TNM	T2aN0M0	T2aN2M0	T2bN1M0	T2aN0M1b	T2aN0M1b	T3N0M1b
Treatment						
Primary	Surgery + CT	Surgery + CT	Surgery + CT	Surgery + CT	CT	Surgery + CT
Metastases	Surgery	Surgery	Surgery	Surgery	Surgery	Surgery

### DNA extraction and exome sequencing

Genomic DNA was extracted from formalin‐fixed, paraffin embedded (FFPE) tissue sections using the QIAamp DNA FFPE Tissue Kit (QIAGEN, Frankfurt, Germany), according to the manufacturer's protocol. DNA was assessed for quality and was qualified using NanoDrop and agarose gel electrophoresis. DNA libraries were created using the SureSelect XT Library Prep Kit (Agilent, Palo Alto, CA, USA), according to the manufacturer's protocol. Exome capture was performed with the Agilent SureSelect kit. The DNA library and exomes were sequenced on the Illumina HiSeq 2500 platform (Illunima, San Diego, CA, USA). The capture and coverage of raw reads were mapped to a human genome reference provided by the Burrows–Wheeler Aligner and reference sequence version HG19 alignment. Indels were called using gatk, and all variants were annotated by annovar.

### Functional and pathway enrichment analysis

sift, lrt and polyphen (polymorphism phenotyping) were used to predict the impact of pathogenic somatic mutations on gene function. The david v6.8 (Database for Annotation, Visualization and Integrated Discovery, https://david.ncifcrf.gov/) was used to analyze the shared pathogenic genes of corresponding primary tumors and metastases, including gene ontology (GO) BP, cellular components (CC) and MF. The Kyoto Encyclopedia of Genes and Genomes (KEGG) pathway was used to examine molecular interactions, reaction and relation networks of the shared pathogenic genes identified from primary tumors and paired metastases. Fisher's exact test was performed; *P* < 0.05 was regarded as indicating statistical significance.

## Results

### Genetic profiling of primary tumors and brain metastases by whole exome sequencing

Exons account for 1% of the human genome but contain approximately 85% of disease‐causing mutations. To investigate the driver alteration genes in brain metastases from NSCLC and potential targeted treatments of driver mutations, we compared 12 samples (primary tumor and matched brain metastases) by exome sequencing. Of these, six were identified by WES, covering 230 418 amplicons of 23 129 genes. Six others were identified by deep targeted next‐generation sequencing of 390 cancer associated genes. WES is a gene analysis method that captures and enriches the entire exonic regions using sequence capture technology and performs high‐throughput sequencing. WES has the advantages of high throughput and low cost, and so we analyzed the whole driver mutant exome profiles in three pairs of specimens using WES.

**Figure 1:**
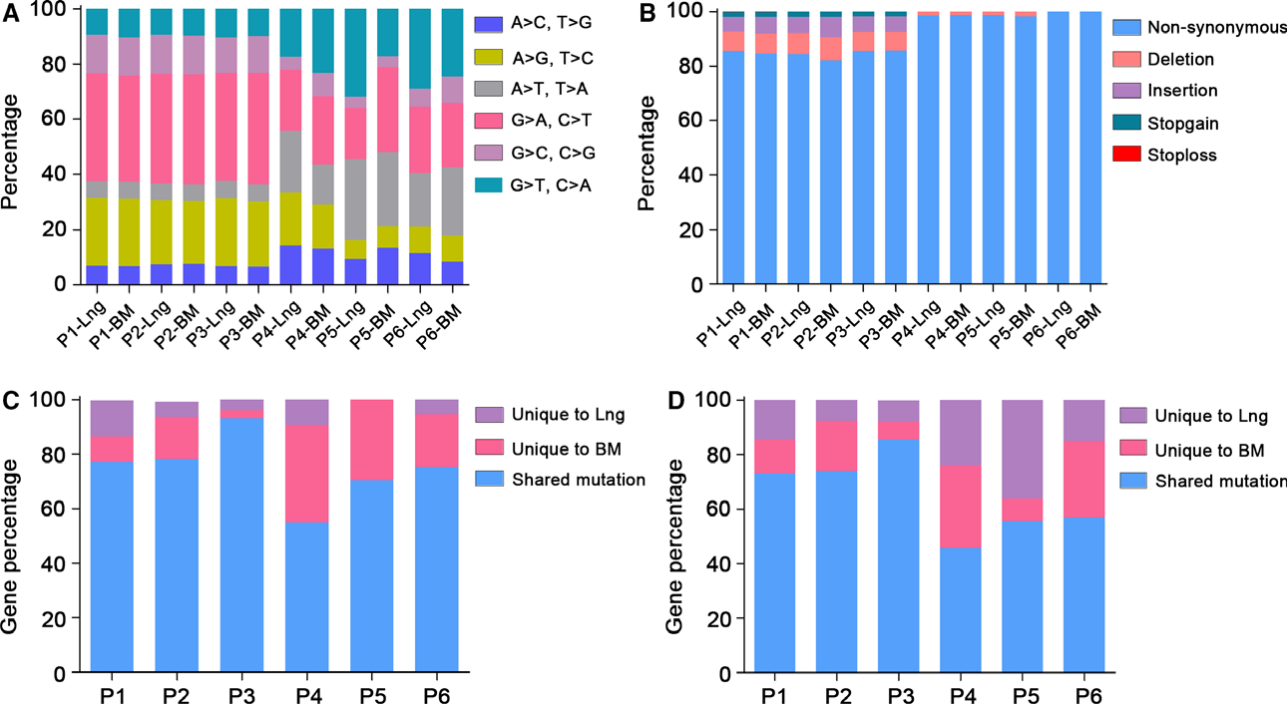
Mutation comparisons of primary and paired‐metastasis tumor samples. (A) The percentage of transversion and transition in each sample. BM, brain metastasis tissue; Lng, primary lung cancer tissue. (B) The proportion of five types of exonic mutations found within each primary and paired‐metastasis tumor sample. Non‐synonymous mutations are shown in blue, deletions in pink, insertions in purple, stop‐gain in green, and stop‐loss in red. (C) The proportion of unique and shared mutant genes in each patient. Bars represent alterations in genes that are unique to primary specimens (purple), brain metastasis specimens (pink), and shared mutation (blue). (D) The proportion of unique and shared mutant SNV in each patient. Bars represent single nucleotide variants that are unique to primary specimens (purple), brain metastasis specimens (pink), and shared mutation (blue).

We carried out WES with an average depth of 375× (range: 323–399×) and identified an average number of 2055 exonic somatic variant sites and 1008 mutant genes in three pairs of primary tumors and corresponding brain metastases. In order to further understand the possibly or probably pathogenic mutations, we used three filtering steps to exclude the volume of non‐pathogenic variants and all variations showing poor quality as follows: (a) read depth < 30, (b) synonymous mutation, and (c) variant frequency < 5%. For P1 primary tumor and matched brain metastasis samples, we identified 2104 (in 1045 genes) and 2052 (in 999 genes) exonic somatic variant sites, respectively, after filtration (Table S1). For P2 primary lung cancer and matched brain metastasis tissues, we identified 1900 (in 949 genes) and 2146 (in 1059 genes) exonic somatic variant sites, respectively, after filtration (Table S2). For P3 primary tumor and matched brain metastasis samples, we identified 2083 (in 1029 genes) and 2044 (in 1017 genes) exonic somatic variant sites, respectively, after filtration (Table S3). Of these, approximately 84.6% were non‐synonymous SNVs, significantly more than the proportion that were deletions (7.4% on average), insertions (6% on average), stop‐gain (1.9% on average), and stop‐loss (less than 0.1% on average) alterations. The transitions A→G, T→C, G→A and C→T were significantly more common than transversions and other types of transitions, but there was a slight difference between primary and paired‐brain metastasis mutations. In this WES cohort, the transition/transversion ratio was 1.73 on average (Fig. 1).

### Genetic profiling of primary tumors and brain metastases by targeted next‐generation sequencing

Compared to WES, a targeted sequencing panel only captures and enriches exonic regions of the targeted genes, but with better detection depth and accuracy. To increase the sensitivity of detection for known targeted gene abnormalities and to decrease the interference of treatments, a targeted sequencing panel was performed in another three untreated patients to identify mutations in genes that may be critical for brain parenchyma metastatic lung cancer. We then performed a deep targeted sequencing panel of 390 cancer‐related genes at an average depth of 678× (range: 370–821×), including 80 targeted treatment genes, 200 genes involved in druggable target‐related signaling pathways, 50 genes involved in DNA damage repair, 25 genes involved in epigenetics, 25 fusion genes, 10 other cancer‐related genes (e.g. *EGFR*,* TP53*,* BRAF*,* PTEN*,* KRAS*, and *BRCA1*). For P4 primary tumor and matched brain metastasis samples, we identified 64 (in 34 genes) and 70 (in 48 genes) exonic somatic variant sites, respectively, after filtration (Table S4). For P5 primary lung cancer and matched brain metastasis tissues, we identified 76 (in 27 genes) and 53 (in 19 genes) exonic somatic variant sites, respectively, after filtration (Table S5). For P6 primary tumor and matched brain metastasis samples, we identified 62 (in 29 genes) and 73 (in 34 genes) exonic somatic variant sites, respectively, after filtration (Table S6). Of these, more than 98% of the mutations were non‐synonymous after filtering. Figure 1 shows a detailed summary of the genetic heterogeneity and homogeneity distribution for all mutations found in each patient.

### Functional prediction and bioinformatic analysis of brain metastasis‐related genes

Non‐synonymous mutations refer to gene mutations that result in altered amino acid sequences of a polypeptide product or altered functional RNA sequences. Because the body has a complex mismatch repair mechanism, not all non‐synonymous mutations are pathogenic or fatal. According to previous studies, we applied sift, lrt and polyphen (polymorphism phenotyping) to further predict the impact of pathogenic somatic mutations on gene function 8, 9. We found that approximately 19.3% of the identified somatic mutations were likely to have pathogenic consequences according to at least two of the three widely accepted methods above. We included mutations annotated as ‘probably damaging’ or ‘possibly damaging’ or ‘deleterious’.

**Figure 2:**
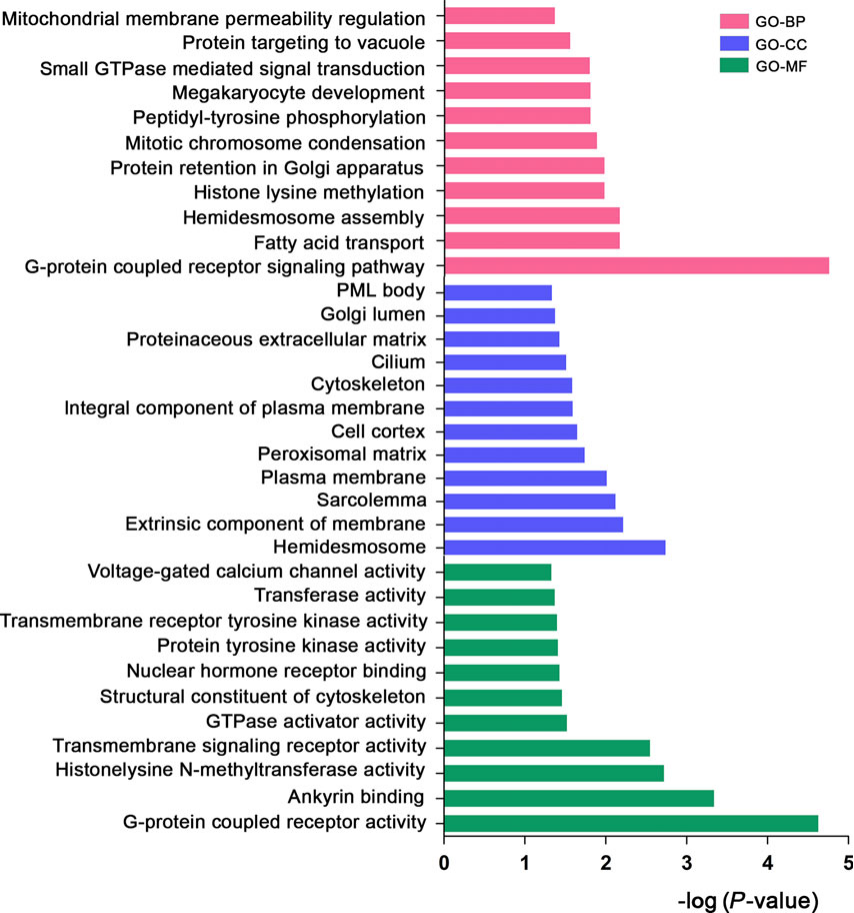
The functional prediction and bioinformatic analysis of shared pathogenic genes of corresponding primary tumors and metastases. Histograms were used to show functional annotation analysis of BP, CC and MF by DAVID v6.8, GO_BP, GO_CC, GO_MF were ranked by −log (*P* value), with a filter of *P* < 0.05 by Fisher's exact test.

In order to better understand the function and pathway enrichment of the possible driver genes for brain metastases in NSCLC, we analyzed 661 commonalities and shared pathogenic genes of corresponding primary tumors and metastases using david (Table S7). We found that the enriched GO BPs and MF were primarily associated with signal transduction and molecular binding (Tables [Link], [Link], [Link]), including G‐protein‐coupled receptor signaling pathway (*n* = 56, *P* = 1.82 × 10^−5^, Fig. 2). We further used the KEGG pathway to examine the molecular interactions, reactions and relational networks of the shared pathogenic genes identified from primary tumors and paired‐brain metastases. After classification, 206 genes were clustered into 126 pathways, of which 52 were associated with cancer‐related signaling pathway (Table S11), including focal adhesion (*COL6A3*,* FLNB*,* LAMC2*,* BAD*,* SHC1*,* LAMA2*,* TNR*, and *EGFR*), ECM‐receptor interactions (*COL6A3*,* LAMC2*,* TNN*,* LAMA2*, and *TNR*), cell cycle (*CDC27*,* MCM2*,* MYT1*, and *EP300*), the VEGF signaling pathway (*PLA2G4A*,* BAD*, and *SPHK2*), the Wnt signaling pathway (*DAAM2*,* WNT10A*, and *EP300*), the TP53 signaling pathway (*BBC3* and *BAI1*), the notch signaling pathway (*NOTCH2*,* NOTCH2NL*,* MAML3*, and *EP300*) and immunity‐associated processes (*CD2*,* HLA*,* CD80*,* NCR2*,* KLRC2*, and *SHC1*).

### Genetic heterogeneity between primary tumors and brain metastases

We compared six paired samples and observed genetic heterogeneity between primary tumor and corresponding brain metastases. Mutations unique to primary tumor or brain metastases may be acquired or lost during malignant progression, suggesting that there may be genetic alterations acquired during malignant progression, possibly participating in BM in NSCLC. The *LDHAL6B*,* CSH1*,* PEX5*, and *YBX2* genes were frequently altered in the primary tumors, as opposed to the brain metastases specimens. The *SLC16A2*,* PLBD2*,* APC*,* ALPPL2*,* SCUBE2*,* OR8G5*, and *EVPL* genes were only mutated in primary tumors but not in brain metastases.

### Concordance of *EGFR* and *TP53* mutations in primary tumors and brain metastases

Four patients had an activating mutation in *EGFR* (L858R or exon 19 deletions) in both the primary tumor and their corresponding BM. Three patients had deletions in exon 19, and one had a mutation in exon 21(L858R). However, T790M was not detected in any of these patients with brain metastases. Additionally, we found that 75% of the patients in our cohort had mutations in *TP53* (R43H, R16H, R136H, and R175H), but no discordance was found in these cases. Of note, the concordance rate between primary NSCLC and brain metastases was 100% (Table 2). *EGFR* and *TP53* status maybe homogeneous in lung cancer tissues and were stable during brain metastases during the malignant process.

**Table 2:** Concordance of *EGFR* and *TP53* mutations in primary tumors and brain metastases

Patient ID	Primary tumor	Brain metastases
P1	*EGFR* deletion in exome 19	*EGFR* deletion in exome 19
P2	*EGFR* L858R *TP53* R43H, R16H, R175H, and R136H	*EGFR* L858R *TP53* R43H, R16H, R175H, and R136H
P3	—	—
P4	*EGFR* deletion in exome 19 *TP53* C135R	*EGFR* deletion in exome 19 *TP53* C135R
P5	*EGFR* deletion in exome 19 *TP53* T86M	*EGFR* deletion in exome 19 *TP53* T86M
P6	*TP53* T23P	*TP53* T23P

### Hotspot mutations in brain‐metastatic NSCLC patients

**Figure 3:**
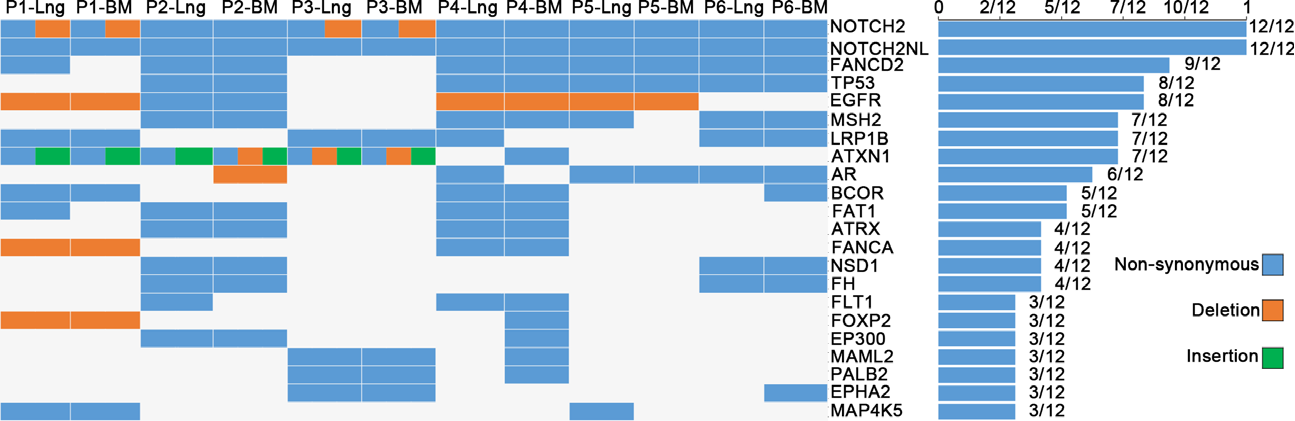
Parts of the most commonly altered cancer‐related genes identified from each specimen. Histograms were ranked by the frequency of significant genes identified from 12 samples. Nonsynonymous mutations are shown in blue, deletions in red, and insertions in green. Lng, primary lung cancer tissue; BM, brain metastases tissue.

We detected some well‐known genes with high‐frequency mutations in 12 tissue samples, including *NOTCH2* (12/12), *NOTCH2NL* (12/12), *FANCD2* (9/12), *TP53* (8/12), and *EGFR* (8/12), followed by *MSH2* (7/12), *ATXN1* (7/12), and *LRP1B* (7/12). Of note, six patients with *NOTCH2* mutations also had *NOTCH2NL* mutations. Figure 3 shows some of the most commonly altered cancer‐related genes identified from each specimen.

## Discussion

Lung cancer is a highly heterogeneous disease that varies in its degree of complexity and heterogeneity during the malignant process. Lung cancer is the most common cancer to metastasize to the brain 10. Given the poor prognosis of brain‐metastatic lung cancer, there is an urgent need to understand brain‐metastatic genetic profiles by comparing the somatic mutations between primary tumors and corresponding brain metastases. Tumor metastasis is a complex and multistage progress, consisting of local invasion, intravasation to the blood or lymphatic system, survival in the circulation, extravasation, and distant colonization 11. Generally, cancer cells have difficulty penetrating the blood–brain barrier with its tight layer of endothelial cells. Nevertheless, lung cancer cells target and infiltrate the brain frequently. Considering this metastatic selective advantage, lung cancer cells may require highly specialized functions during infiltration into the brain parenchyma 12. The genetic alterations driving brain organ‐specific metastases remain unknown.

Previous efforts have been made to characterize the genomic heterogeneity of several target genes between primary lung cancer and brain metastases, but the driver mutant genetic profiles and underlying mechanisms of BM progress remain poorly understood. For example, Fan *et al*. 13 performed next‐generation targeted panel sequencing of paired primary tumor and corresponding cerebrospinal fluid samples from 11 *EGFR* mutation‐positive adenocarcinoma patients with leptomeningeal metastases. They showed a high degree of genetic heterogeneity and divergence within primary tumors and corresponding cerebrospinal fluid samples. Mansfield *et al*. 14 analyzed the expression of programmed cell death ligand 1 (*PD‐L1*) in paired lung cancer and brain metastases from 73 patients using immunohistochemistry and showed that there was an agreement of *PD‐L1* expression in 86% of the paired specimens. However, Pinato *et al*.'s 15 and Zhou *et al*.'s 16 studies suggested a spatial and temporal heterogeneity in the expression of *PD‐L1* between isogeneic NSCLC and corresponding distant metastases, and they believed that such heterogeneity was significantly dependent on whether BM was synchronously diagnosed along with primary disease.

Our data suggested that the detected SNVs and associated mutant genes varied markedly not only within paired primary tumor and corresponding brain metastases but also in different patients. Intratumor heterogeneity may be influenced by chromosome instability, and therefore metastasis may be driven via selection of cancer cells with SNV and may increase the risk of recurrence or death 17. Genetic heterogeneity acquired during malignant progression highlights the challenge of exploring the mechanisms of BM. Additional genetic studies and functional assessments are needed to explore the contribution of mutant molecular profiles and their role in brain metastases.

Even though we observed high genetic heterogeneity in paired primary tumors and brain parenchyma metastases, we found that mutations in *EGFR* and *TP53* were highly concordant between paired primary tumors and brain metastases (100% concordance), the same as the concordance reported by Matsumoto *et al*. 18. The majority of previous studies demonstrated that the overall concordance rate of *EGFR* mutation status was about 87.8% between primary tumor and paired metastatic lymph nodes 19. A higher concordance rate was observed in Sun *et al*.'s 20 and Schmid *et al*.'s 21 studies (91% and 94%, respectively). Yatabe *et al*. 22 reported no divergent *EGFR* mutations among 77 paired primary and matched lymph node metastases or among 54 primary and corresponding recurrent tumors using RT‐PCR. Fan *et al*. 13 reported that *EGFR* mutations detected in the primary adenocarcinoma were highly concordant with those of matched cerebrospinal fluid samples from patients with brain metastases. Luo *et al*. 23 analyzed the *EGFR* mutational status in NSCLC patients and found a concordance rate of 93.3% between 15 paired primary and brain metastasis samples by an amplification mutation refractory system. Shimizu *et al*. 24 found that *EGFR* mutations detected in all metastatic sites were also detected in the corresponding primary tumors by the peptide nucleic acid‐locked nucleic acid polymerase chain reaction, and that *EGFR* mutations in metastasis sites were sensitive markers of the response to targeted therapy in NSCLC patients. The slight discordance rate of mutations between paired primary tumors and metastases may be partly influenced by the testing technology 25 and sample quality 26. Thus, our results agree with previously published data. The implication is that the stable *EGFR* mutational status between paired primary tumors and metastatic sites confirms that most mutations detected on analysis of the primary tumor or metastases are sufficient for clinical decision‐making and that there is no need to re‐biopsy recurrent tumors or metastases for most of the NSCLC patients.

*EGFR* mutations can be detected in more than 40% of the East Asian population with NSCLC 10. In our experience, *EGFR*‐activating mutations were detected in four of six patients, including L858R in one patient and exon 19 deletion in three patients. However, T790M was not detected in any of these patients with brain metastases. Additionally, *EGFR* was most frequently mutated in NSCLC, reported by COSMIC (approximately 27%). By comparing *EGFR* mutations with those reported in COSMIC, we identified a higher prevalence of *EGFR* mutations in patients with brain metastases. Similarly, Matsumoto *et al*. 18 reported *EGFR* mutations in 61% of lung adenocarcinoma patients with brain metastases. Based on previous studies and our group's previous study, we conclude that patients with brain metastases harbor *EGFR* mutations more frequently than do those without, and that *EGFR* mutations are poor prognostic factors in stage IV patients with brain metastases 27.

Additionally, we found that 75% of the specimens in our cohort had mutations in *TP53*. However, the result is limited by the small number of patients; more studies and functional assessments are required. The concordance within the paired samples is also noteworthy, as *TP53* could be a potential therapeutic target for brain metastases. The well‐known tumor suppressor gene *TP53* participates in inhibition of the propagation of cancer cells with genome instability 28. Therefore, mutations in *TP53* may facilitate chromosome‐unstable cell proliferation without regulation. Previous sequencing studies investigated *TP53* molecular characteristics in NSCLC within primary tumors and between primary tumors and metastases 29. Multi‐region WES was performed in 100 NSCLC patients, revealing that driver mutations in *EGFR* and *TP53* were almost exclusively clonal in different primary tumor regions from the same patients 17. Other studies and reviews also showed that intratumoral heterogeneity of *TP53* mutations was rare in NSCLC and corresponding metastases 30.

We also identified a large number of *NOTCH2*,* NOTCH2NL*, and *FANCD2* mutations, much higher than the mutation rates reported by COSMIC in NSCLC (3%, 0.64%, and 0.94%, respectively). Of note, six patients with *NOTCH2* mutations also had *NOTCH2NL* mutations. However, the result is limited by the small number of patients. More studies and functional assessments are required.

## Conclusion

We observed genetic heterogeneity not only between the primary tumors and metastases, but also in different patients. These results imply that most genetic alterations may be acquired or lost during malignant progression. However, mutations in *EGFR* and *TP53* remained clonal during the malignant process. Concordance mutations in *EGFR* and *TP53* suggest that activated oncogene *EGFR* and disrupted tumor suppressor *TP53* may contribute to brain metastases. *EGFR* and *TP53* mutations detected from the primary tumor may be sufficient for clinical treatment decision‐making, and additional *EGFR* and *TP53* testing of brain metastases may be unnecessary.

## Author contributions

XZ and XL conceived the study design. MG, RH and QZ collected the human samples. LL and XJ performed the analysis. LL wrote the paper. All authors read and approved the final manuscript.

## Supporting information


**Table S1.** Mutant genes identified in P1 primary tumor and matched brain metastasis samples.

**Table S2.** Mutant genes identified in P2 primary tumor and matched brain metastasis samples.

**Table S3.** Mutant genes identified in P3 primary tumor and matched brain metastasis samples.

**Table S4.** Mutant genes identified in P4 primary tumor and matched brain metastasis samples.

**Table S5.** Mutant genes identified in P5 primary tumor and matched brain metastasis samples.

**Table S6.** Mutant genes identified in P6 primar tumors and matched brain metastasis samples.

**Table S7.** Shared pathogenic genes of six corresponding primary tumors and brain metastases (661 in total).

**Table S8.** The gene ontology biological processes (GO‐BP) analysis of shared pathogenic genes of corresponding primary tumors and metastases.

**Table S9.** The gene ontology cellular component (GO‐CC) analysis of shared pathogenic genes of corresponding primary tumors and metastases.

**Table S10.** The gene ontology molecular function (GO‐MF) analysis of shared pathogenic genes of corresponding primary tumors and metastases.

**Table S11.** KEGG pathway analysis: 52 pathways associated with cancer‐related signaling.
