# Supplementary material for: Characterization of genetic alterations in brain metastases from non‐small cell lung cancer
Source: FEBS Open Bio. 2018 Aug 30;8(9):1544–52. doi: 10.1002/2211-5463.12501 (PMC6120240; doi:10.1002/2211-5463.12501)
Supplement: Supplementary file 1 — Table S1. Mutant genes identified in P1 primary tumor and matched brain metastasis samples. [file FEB4-8-1544-s001.docx]

**Supplemental table 1. Mutant genes identified in P1 primary tumors and matched brain metastases samples.**

| **P1- primary tumors** | **P1- brain metastases** |
| --- | --- |
| PDE4DIP | NBPF8 |
| MST1L | OR2T35 |
| CDK11A, CDK11B | PDE4DIP |
| NOTCH2NL | NOTCH2NL |
| DDAH1 | OR2T3 |
| OR2T3 | HNRNPCL1, HNRNPCL3, HNRNPCL4 |
| OR2T34 | OR2T34 |
| HNRNPCL1, HNRNPCL3, HNRNPCL4 | MST1L |
| ERO1LB | FAM159A |
| HRNR | HRNR |
| NBPF8 | FLG |
| OR2T1 | CEP170 |
| CCT3 | FCGR3A |
| CEP170 | CCT3 |
| FCGR3A | CDK11A, CDK11B |
| RPTN | OR2T2 |
| PPIAL4G | PPIAL4G |
| OR2T35 | GPSM2 |
| FLG | RPTN |
| PDC | OR2T1 |
| OR2L2 | NBPF10 |
| OR2T2 | CALML6 |
| CHML | NBPF4 |
| NBPF10 | DDAH1 |
| MPL | ERO1LB |
| VPS13D | PER3 |
| PRAMEF2 | OR2L2 |
| GPSM2 | MPL |
| SLAMF8 | HNRNPCL2 |
| PER3 | PRAMEF1 |
| CNST | PDC |
| PRDM2 | HMCN1 |
| TAS1R1 | SLAMF8 |
| OR4F5 | CHML |
| HNRNPCL2 | TAS1R1 |
| LUZP1 | VPS13D |
| PMF1, PMF1-BGLAP | PRDM2 |
| COA6 | F13B |
| RPE65 | GJA5 |
| DFFB | PRAMEF2 |
| RHD | TPR |
| PRAMEF1 | MAGI3 |
| COL24A1 | RHD |
| NBPF4 | VTCN1 |
| CENPF | PTCHD2 |
| AXDND1 | COA6 |
| PLA2G4A | DFFB |
| MFSD2A | CENPF |
| CALML6 | OR4F5 |
| VTCN1 | OR2T29 |
| EIF2B3 | PMF1, PMF1-BGLAP |
| MTF2 | CNST |
| OR2T27 | CTPS1 |
| MTOR | LAMC1 |
| IGFN1 | RPE65 |
| OR2T29 | IGFN1 |
| FCRLA | FCRLA |
| OR2T5 | MTFR1L |
| NADK | EIF2B3 |
| SUSD4 | ANKRD35 |
| OR2T12 | OR2T27 |
| EVI5 | BMP8B |
| ATP8B2 | OR2T5 |
| HSPG2 | CSMD2 |
| BTG2 | ATP8B2 |
| LAMC2 | MTOR |
| TARBP1 | MTF2 |
| CSMD2 | OXCT2 |
| LAMC1 | COL24A1 |
| MACF1 | GPR137B |
| ANKRD35 | ARV1 |
| BMP8B | NADK |
| TCHH | HSPG2 |
| MTHFR | AK2 |
| OR2T8 | OR2T12 |
| OXCT2 | EPHX1 |
| NOTCH2 | LUZP1 |
| GPR137B | BTG2 |
| NBPF14, NBPF8, NBPF9 | LAMC2 |
| DUSP12 | MFSD2A |
| LEPRE1 | NBPF14, NBPF8, NBPF9 |
| LAD1 | OR2T8 |
| RGS4 | MTHFR |
| MYOC | SUSD4 |
| MIR205HG | TCHH |
| SPRR3 | AXDND1 |
| ITLN1 | SLC35E2 |
| SLC35E2 | NOTCH2 |
| EFNA4 | TARBP1 |
| OBSCN | PRAMEF4 |
| KDF1 | MACF1 |
| PLEKHM2 | NPL |
| EPHX1 | KDF1 |
| UBXN11 | CDCP2 |
| CASZ1 | CASZ1 |
| CDCP2 | EFNA4 |
| MRPL24 | LAD1 |
| CTSE | PLEKHM2 |
| ZSWIM5 | EVI5 |
| FAIM3 | PLA2G4A |
| NBPF15 | OBSCN |
| HIVEP3 | ITLN1 |
| KCNN3 | DUSP12 |
| LOC101929983, PRAMEF6, PRAMEF9 | RGS4 |
| TMEM51 | LEPRE1 |
| CROCC | MRPL24 |
| AIM1L | TMEM51 |
| PRAMEF4 | LOC101929983, PRAMEF6, PRAMEF9 |
| LCE1D | CTSE |
| LOC391003, PRAMEF22 | AIM1L |
| BMP8A | KCNN3 |
| BEND5 | ZSWIM5 |
| PRG4 | MYOC |
| KIAA0040 | UBXN11 |
| OR2T33 | BMP8A |
| AGRN | BEND5 |
| ATAD3B | HIVEP3 |
| FBXO2 | MIR205HG |
| LOC100996758, NPY4R | PRAMEF12 |
| WAC | FAIM3 |
| SYT15 | CDK11A |
| ACBD5 | NBPF15 |
| SVIL | LOC391003, PRAMEF22 |
| GPRIN2 | KIAA0040 |
| LYZL2 | CROCC |
| TIMM23 | LRRC71 |
| IDE | OR2T33 |
| SEMA4G | FBXO2 |
| ZNF239 | LCE1D |
| FRG2B | FOXO6 |
| ZFYVE27 | AGRN |
| KAT6B | FAM43B |
| THNSL1 | LOC100996758, NPY4R |
| NRAP | SYT15 |
| DMBT1 | GPRIN2 |
| ABI1 | LYZL2 |
| AFAP1L2 | WAC |
| TACC2 | KAT6B |
| C10orf90 | NRAP |
| EIF5AL1 | SEMA4G |
| ADAMTS14 | SVIL |
| LRRC18 | ACBD5 |
| KIF11 | DMBT1 |
| BMS1 | FRG2B |
| SFRP5 | ABI1 |
| IDI1 | TIMM23 |
| PNLIP | LRRC18 |
| KCNMA1 | TACC2 |
| STK32C | EIF5AL1 |
| LZTS2 | AFAP1L2 |
| BTAF1 | C10orf90 |
| FAM178A | PNLIP |
| OR8U1, OR8U8 | IDE |
| OR9G1, OR9G9 | MYO3A |
| OR4C3 | ZFYVE27 |
| OR8B2 | ADAMTS14 |
| MUC6 | BMS1 |
| OR51B5 | SFRP5 |
| NLRP14 | KCNMA1 |
| SAA2, SAA2-SAA4 | STK32C |
| CNTN5 | IDI1 |
| SERPING1 | KIF11 |
| OR8D2 | CHST15 |
| MICALCL | FZD8 |
| OR8U1, OR8U8 | OR8U1, OR8U8 |
| CEP295 | OR52E4 |
| OR51F1 | OR4C3 |
| OR10G4 | MUC5B |
| CDON | OR9G1, OR9G9 |
| LRP4 | OR8B2 |
| UPK2 | MUC6 |
| PGR | OR8G1, OR8G5 |
| TENM4 | OR10G4 |
| HPS5 | SAA2, SAA2-SAA4 |
| ROBO4 | PGR |
| OR8U1 | NLRP14 |
| OR10G7 | OR8G5 |
| C11orf40 | OR51F1 |
| DNHD1 | OR8D2 |
| KDM4E | OR8U1, OR8U8 |
| MUC5B | HPS5 |
| C11orf80 | KRTAP5-3 |
| CRY2 | TMPRSS13 |
| MUC2 | SERPING1 |
| SHANK2 | CEP295 |
| FERMT3 | SHANK2 |
| ZNF215 | KRTAP5-7 |
| KRTAP5-5 | LRP4 |
| NAV2 | RASSF7 |
| HTR3A | HTR3A |
| TMPRSS13 | CDON |
| ANO5 | DNHD1 |
| ESRRA | KDM4E |
| OR1S1 | ROBO4 |
| TRPM5 | TRPM5 |
| DCHS1 | UPK2 |
| GAL3ST3 | MTCH2 |
| TRIM49C | C11orf40 |
| RASSF7 | OR10G7 |
| MYRF | CRY2 |
| KRTAP5-7 | NAV2 |
| IFITM10 | OR1S1 |
| NRIP3 | MUC2 |
| MAP4K2 | C11orf80 |
| KRTAP5-4 | OTOG |
| OTOG | ESRRA |
| SAA1 | TRIM49C |
| KCNC2 | ANO5 |
| EEA1 | MICALCL |
| TAS2R31 | DCHS1 |
| TAS2R46 | KRTAP5-4 |
| OTOGL | DAGLA |
| FGD6 | IFITM10 |
| C12orf79 | MAP4K2 |
| TAS2R30 | SAA1 |
| TAS2R19 | SCUBE2 |
| SLC2A3 | DGKZ |
| TCP11L2 | KCNC2 |
| KLRC2 | TAS2R46 |
| C1RL | SLC2A3 |
| TAS2R43 | OTOGL |
| PHLDA1 | TAS2R31 |
| IKBIP | TAS2R30 |
| TUBA1C | KLRC2 |
| MGP | C12orf79 |
| DCP1B | TAS2R19 |
| OR10AD1 | FGD6 |
| ABCB9 | C1RL |
| KRT6B | TUBA1C |
| CNOT2 | DCP1B |
| OAS2 | PHLDA1 |
| PSMD9 | ATN1 |
| SDR9C7 | PSMD9 |
| CACNA2D4 | ABCB9 |
| C12orf42 | SDR9C7 |
| ATN1 | CELA1 |
| ZCCHC8 | KRT6B |
| CELA1 | PRH2 |
| ALDH1L2 | TCP11L2 |
| SLC6A15 | ZNF384 |
| DDX51 | ZCCHC8 |
| PRH2 | P2RX7 |
| IRAK4 | C12orf42 |
| C12orf60 | PRB1 |
| MYO1H | TAS2R43 |
| FAM186A | ALDH1L2 |
| CAMKK2 | FAM186A |
| PRB1 | CAMKK2 |
| KLRC3 | IRAK4 |
| WDR66 | SLC6A15 |
| RNFT2 | KLRC3 |
| KRT2 | RNFT2 |
| PLBD1 | KMT2D |
| KRT18 | WDR66 |
| CEP290 | NOC4L |
| NOC4L | KRT2 |
| ASCL1 | GXYLT1 |
| BRI3BP | DDX51 |
| HCAR3 | KRT18 |
| KMT2D | PLBD2 |
| TPTE2 | HCAR3 |
| VWA8 | ASCL1 |
| PABPC3 | CEP290 |
| C1QTNF9 | TPTE2 |
| SKA3 | C1QTNF9 |
| PSPC1 | PSPC1 |
| BIVM, BIVM-ERCC5 | BIVM, BIVM-ERCC5 |
| STARD13 | VWA8 |
| LMO7 | STARD13 |
| LRRC63 | SKA3 |
| PARP4 | PABPC3 |
| MTUS2 | MTUS2 |
| TRPC4 | PARP4 |
| LRCH1 | LRCH1 |
| ZIC5 | ZIC5 |
| EPSTI1 | SOX1 |
| PIBF1 | DACH1 |
| DACH1 | PIBF1 |
| WDR89 | EPSTI1 |
| RNF212B | RNF212B |
| RBM23 | WDR89 |
| SYNE2 | RBM23 |
| PAPLN | SYNE2 |
| TEP1 | OR4K2 |
| OR4K2 | SLC10A1 |
| RIN3 | NID2 |
| MAP4K5 | AHNAK2 |
| SLC10A1 | MYH6 |
| NID2 | POTEG |
| AHNAK2 | OXA1L |
| HOMEZ | REC8 |
| RAB15 | RAB15 |
| REC8 | RIN3 |
| MYH6 | OR4N2 |
| RTL1 | MAP4K5 |
| OXA1L | TMEM121 |
| OR4N2 | EXOC3L4 |
| POTEG | ADAM21 |
| IFI27L2 | MAP3K9 |
| EXOC3L4 | HOMEZ |
| TMEM121 | NRDE2 |
| ADAM21 | OR10G2 |
| NRDE2 | OR4M2 |
| CCDC88C | LINS |
| OR4M2 | ANKRD34C |
| LINS | TJP1 |
| ZSCAN2 | PHGR1 |
| PHGR1 | ADAMTS17 |
| TJP1 | ZSCAN2 |
| PLIN1 | GOLGA8R |
| LRRK1 | FAM154B |
| LDHAL6B | MEF2A |
| ANKRD34C | OR4N4 |
| MESDC2 | ZNF280D |
| GOLGA8R | PML |
| OR4N4 | MESDC2 |
| PEAK1 | HERC2 |
| ADAMTS17 | LRRK1 |
| FAM154B | NPAP1 |
| C15orf40 | ADAMTS7 |
| MEF2A | GOLGA8A |
| ANPEP | LOC283710 |
| MAPKBP1 | CYP11A1 |
| NPAP1 | CHRNA3 |
| HERC2 | SMAD6 |
| PML | PDXDC1 |
| ADAMTS7 | SPIRE2 |
| TLN2 | OTOA |
| LOC283710 | PDPR |
| TMED3 | C16orf46 |
| CYP11A1 | ZNF500 |
| CHRNA3 | KDM8 |
| ACSM2B | FANCA |
| PDXDC1 | ZFHX3 |
| FANCA | C16orf93 |
| C16orf46 | GLYR1 |
| CNGB1 | HYDIN |
| CES1 | CLEC18C |
| SLC12A4 | E2F4 |
| SPIRE2 | NARFL |
| ZFHX3 | ACSM2B |
| OTOA | SULT1A1 |
| C16orf93 | CLEC18B |
| VWA3A | SRRM2 |
| SSTR5 | PKD1L2 |
| HYDIN | TPSAB1 |
| COG8 | TPSD1 |
| SNAI3 | TPSB2 |
| IL32 | GSPT1 |
| CLEC18B | CNOT1 |
| NARFL | IFT140 |
| SULT1A1 | IRX6 |
| GLYR1 | FBXW10 |
| GSPT1 | KCNJ12, KCNJ18 |
| PKD1L2 | SARM1 |
| ZNF500 | MAP2K3 |
| BEAN1 | LRRC37A3 |
| CLEC18C | TNRC6C |
| TPSAB1 | CCDC144NL |
| SMPD3 | MYO18A |
| PHLPP2 | CPD |
| TPSD1 | EFCAB5 |
| MTHFSD | PIGW |
| TPSB2 | NFE2L1 |
| IFT140 | 10-Mar |
| TNRC6C | CNTROB |
| FBXW10 | CNP |
| CPD | NTN1 |
| EFCAB5 | USP6 |
| KCNJ16 | DHRS7C |
| LRRC37A3 | FBXO47 |
| KCNJ12, KCNJ18 | RNF43 |
| MAP2K3 | SPPL2C |
| HSF5 | NLRP1 |
| SP2 | FADS6 |
| SARM1 | SLC16A6 |
| INTS2 | KRT15 |
| NFE2L1 | QRICH2 |
| TNS4 | MIEF2 |
| ABCA8 | ABCA8 |
| PIGW | TBC1D16 |
| FBXO47 | KRTAP4-1 |
| TMEM102 | TBC1D3B, TBC1D3F |
| SLC16A6 | PER1 |
| 10-Mar | SCIMP |
| CNTROB | MPRIP |
| USP6 | INCA1 |
| MYO18A | ENO3 |
| NTN1 | CDC27 |
| NCOR1 | NCOR1 |
| SCIMP | GPR142 |
| CNP | OR1D5 |
| INCA1 | CCDC57 |
| HAP1 | RAI1 |
| BPTF | KRTAP9-2 |
| ABCA10 | ICT1 |
| NLRP1 | MYBBP1A |
| KRTAP1-3 | SLC25A10 |
| RAI1 | TBC1D28 |
| KRT37 | SPDYE4 |
| PLEKHH3 | ABCA10 |
| KRT15 | BPTF |
| RNF43 | KDM6B |
| PER1 | LRRC37A |
| MPRIP | GSDMA |
| ICT1 | PLXDC1 |
| MIEF2 | KCNAB3 |
| FADS6 | RILP |
| DHRS7C | TRIM16 |
| QRICH2 | USP36 |
| USP43 | TRIM65 |
| SPPL2C | CCDC102B |
| TBC1D16 | FAM69C |
| CDC27 | TAF4B |
| ENO3 | CNDP1 |
| TBC1D3B, TBC1D3F | LPIN2 |
| KRTAP4-1 | POTEC |
| MYADML2 | MYO5B |
| YBX2 | DLGAP1 |
| SLC6A4 | EPG5 |
| LOC100506388 | MBD1 |
| GPR142 | ANKRD30B |
| KDM6B | LILRA6, LILRB3 |
| SPDYE4 | RFPL4AL1 |
| PLXDC1 | ZNF417 |
| CCDC57 | URI1 |
| KRTAP9-2 | ZNF302 |
| LRRC37A | ZNF562 |
| TRIM16 | ZNF780B |
| MYH2 | PEG3 |
| GSDMA | SIGLEC11 |
| CSH1 | RFPL4A |
| USP36 | MUC16 |
| AANAT | PLIN4 |
| RILP | NLRP11 |
| OR1D5 | ZNF814 |
| TXNDC2 | NWD1 |
| CCDC102B | KIR2DL1 |
| LPIN2 | WDR87 |
| TAF4B | ANKRD27 |
| EPG5 | NAPSA |
| ANKRD30B | ZNF181 |
| POTEC | ZNF285 |
| CNDP1 | GP6 |
| MBD1 | HMHA1 |
| ZNF417 | KIAA1683 |
| URI1 | RYR1 |
| ZNF302 | ZNF134 |
| RFPL4AL1 | ZNF440 |
| ZNF780B | ALDH16A1 |
| PEG3 | ZNF135 |
| ZNF562 | KRI1 |
| RFPL4A | MED26 |
| LILRA6, LILRB3 | ZNF443 |
| WDR87 | LILRB1 |
| NAPSA | FCGBP |
| ZNF814 | FFAR3 |
| NLRP11 | ZNF544 |
| MUC16 | RDH13 |
| ZNF570 | DUS3L |
| ZNF676 | B3GNT3 |
| SIPA1L3 | ZNF587 |
| ANKRD27 | CCDC151 |
| SIGLEC11 | MAST1 |
| ZNF181 | LPPR2 |
| ZNF440 | TYK2 |
| NWD1 | THOP1 |
| RYR1 | SLC8A2 |
| PLIN4 | HRC |
| TJP3 | ZNF223 |
| ZNF135 | MED16 |
| HMHA1 | ZNF83 |
| ZNF587 | ANKLE1 |
| ALDH16A1 | HSPBP1 |
| ZNF285 | CLEC4G |
| CYP4F12 | PLA2G4C |
| KRI1 | KMT2B |
| ZNF304 | PLEKHA4 |
| KIR2DL1 | KCNN1 |
| MAST1 | SIGLEC10 |
| FCGBP | LOC100129083, SIGLEC10 |
| ADM5 | MADCAM1 |
| CCDC151 | CEBPA |
| GP6 | KIR2DS1 |
| FFAR3 | KIR3DS1 |
| MED26 | KIR2DS3, KIR2DS5 |
| ZNF223 | KIR2DL5A, KIR2DL5B |
| ZNF83 | KIR2DL2 |
| LPPR2 | TSPYL6 |
| LILRB1 | COL6A3 |
| SLC8A2 | LRP1B |
| KMT2B | AGAP1 |
| RDH13 | UGT1A3 |
| DUS3L | UGT1A5 |
| KIAA1683 | EGR4 |
| ZNF544 | SCTR |
| SLC1A5 | IMP4 |
| HRC | TANC1 |
| HSPBP1 | ST6GAL2 |
| KCNN1 | MYO7B |
| CLEC4G | ANKRD36 |
| PLEKHA4 | SPATA3 |
| TYK2 | CCT7 |
| ANKLE1 | GALNT14 |
| PLA2G4C | SLC8A1 |
| MADCAM1 | IRS1 |
| REXO1 | CPS1 |
| KIR2DL2 | USP37 |
| KIR3DS1 | OR6B3 |
| KIR2DS1 | TTC7A |
| KIR2DS3, KIR2DS5 | TTN |
| KIR2DL5A, KIR2DL5B | WNT10A |
| ANKRD36 | TUBA3E |
| RTN4 | KCNF1 |
| LRP1B | HOXD9 |
| COL6A3 | CTNNA2 |
| CCT7 | REG3A |
| TSPYL6 | RIF1 |
| SPATA3 | LRRTM1 |
| AGAP1 | PARD3B |
| SSFA2 | HS6ST1 |
| CPS1 | VIT |
| M1AP | GCKR |
| SCTR | POTEJ |
| TANC1 | CCDC74A |
| MYO7B | POTEF |
| SLC8A1 | RBM45 |
| USP37 | CAPN13 |
| TTN | ALPPL2 |
| ST6GAL2 | PIKFYVE |
| UGT1A5 | SLC11A1 |
| IRS1 | POTEE |
| EGR4 | MTX2 |
| C2orf71 | AFF3 |
| WNT10A | CCDC74B |
| IMP4 | TTC30A |
| PARD3B | FOXD4L1 |
| RIF1 | TUBA3D |
| HOXD9 | PLCB4 |
| KCNF1 | CRNKL1 |
| CERKL | ADNP |
| TUBA3E | FAM83C |
| CAPN10 | AP5S1 |
| EPCAM | TLDC2 |
| VIT | NCOA3 |
| LRRTM1 | ZMYND8 |
| RBM45 | ARHGAP40 |
| TTC7A | HCK |
| GCKR | PROKR2 |
| SFTPB | LAMA5 |
| REG3A | MIR1-1HG |
| CAPN13 | DEFB132 |
| C2orf73 | CHRNA4 |
| MTX2 | LZTS3 |
| PIKFYVE | CFAP61 |
| ALPP | PHF20 |
| OR6B3 | OCSTAMP |
| POTEF | SIRPB1 |
| CCDC74A | TGM2 |
| FOXD4L1 | CEP250 |
| SLC11A1 | CTSA |
| POTEJ | ZGPAT |
| TTC30A | RALY |
| AFF3 | NPEPL1 |
| CRNKL1 | SIRPA |
| ADNP | TPTE |
| FAM83C | BAGE2, BAGE3 |
| ARHGAP40 | KRTAP10-1 |
| CFAP61 | TMPRSS2 |
| ZMYND8 | KRTAP10-7 |
| HCK | BAGE, BAGE4, BAGE5 |
| PLCB4 | KRTAP10-10 |
| NCOA3 | KRTAP10-2 |
| PROKR2 | PFKL |
| DEFB132 | C21orf58 |
| LAMA5 | KRTAP10-4 |
| PHF20 | URB1 |
| TLDC2 | KRTAP19-7 |
| AP5S1 | FTCD |
| CEP250 | COL18A1 |
| TGM2 | SH3BGR |
| OCSTAMP | KRTAP10-6 |
| MIR1-1HG | CXADR |
| SIRPB1 | LGALS2 |
| CTSA | CCT8L2 |
| ZGPAT | GTSE1 |
| LZTS3 | RFPL3 |
| RALY | TANGO2 |
| NPEPL1 | UPB1 |
| BAGE2, BAGE3 | OSBP2 |
| TPTE | TRMT2A |
| BAGE, BAGE4, BAGE5 | PIM3 |
| TMPRSS2 | TRMU |
| URB1 | NEFH |
| KRTAP10-1 | SUSD2 |
| KRTAP10-7 | SERPIND1 |
| C21orf58 | UPK3A |
| KRTAP19-7 | TRIOBP |
| KRTAP10-10 | BPIFC |
| PFKL | TCF20 |
| KRTAP10-4 | MED15 |
| SH3BGR | POTEH |
| FTCD | BAIAP2L2 |
| COL18A1 | CDC42EP1 |
| LGALS2 | TTLL12 |
| CCT8L2 | SRRD |
| UPB1 | MUC20 |
| RFPL3 | ACKR4 |
| PIM3 | C3orf30 |
| OSBP2 | SLC2A2 |
| NEFH | OXNAD1 |
| TANGO2 | LNP1 |
| UPK3A | HTR1F |
| CYP2D6 | WDR6 |
| TRIOBP | POMGNT2 |
| GTSE1 | EFHB |
| SERPIND1 | COL6A5 |
| MED15 | ZBBX |
| TRMU | SIDT1 |
| BPIFC | CCDC14 |
| TCF20 | CACNA1D |
| SUSD2 | TRAK1 |
| BAIAP2L2 | SLC22A13 |
| CDC42EP1 | KIAA2018 |
| SRRD | DAG1 |
| POTEH | PLXND1 |
| TTLL12 | SRGAP3 |
| APOBEC3A | HEG1 |
| MUC20 | ZNF717 |
| EFHB | MAGEF1 |
| C3orf17 | ABTB1 |
| ATP13A5 | NR1I2 |
| OXNAD1 | IQSEC1 |
| ACKR4 | PLCXD2 |
| SLC2A2 | GADL1 |
| HTR1F | MUC4 |
| VPS8 | KCNMB3 |
| KIAA1407 | TMEM43 |
| KNG1 | RBM5 |
| COL6A5 | RPL14 |
| ZBBX | PTPN23 |
| TOPBP1 | P4HTM |
| C3orf30 | FLNB |
| CEP97 | GOLIM4 |
| WDR6 | LSMEM2 |
| GADL1 | SLC6A11 |
| CACNA1D | NKTR |
| CCDC14 | BSN |
| SLC22A13 | CCDC66 |
| SIDT1 | ERICH6 |
| LNP1 | TMIE |
| ACOX2 | MAGI1 |
| FANCD2 | YEATS2 |
| MAGEF1 | TREX1 |
| DAG1 | MANF |
| GOLIM4 | ATP13A3 |
| RPL14 | FRG1 |
| KIAA2018 | TLR10 |
| PLCXD2 | PPEF2 |
| TRAK1 | APBB2 |
| POMGNT2 | OTOP1 |
| EPHB1 | C4orf26 |
| ZNF717 | ZFYVE28 |
| CCDC66 | NAF1 |
| NKTR | TLR1 |
| HEG1 | KLHL2 |
| PTPN23 | GALNTL6 |
| GLB1 | WFS1 |
| PLXND1 | TRIM60 |
| MAATS1 | RNF212 |
| IL17RC | LYAR |
| TREX1 | TDO2 |
| KCNMB3 | ASIC5 |
| TMEM43 | MAML3 |
| NR1I2 | DSPP |
| IQSEC1 | FAM160A1 |
| MAGI1 | BMP2K |
| ABTB1 | POU4F2 |
| FLNB | CEP135 |
| SRGAP3 | EVC |
| MUC4 | TMEM156 |
| RBM5 | DCHS2 |
| SLC6A11 | COMMD8 |
| ERICH6 | HTT |
| NISCH | SDHA |
| TMIE | ZDHHC11 |
| LSMEM2 | C5orf60 |
| YEATS2 | MAP3K1 |
| FAM157A | PCDHGB4 |
| P4HTM | NOP16 |
| MST1 | LRRC70 |
| BSN | SEC24A |
| FRG1 | BTNL8 |
| C4orf26 | SLC26A2 |
| SYNPO2 | PCDHA4 |
| TLR10 | MYOT |
| NAF1 | BRD9 |
| APBB2 | GPRIN1 |
| TLR1 | TRIM23 |
| TRIM60 | ARHGEF28 |
| KLHL2 | IL9 |
| LYAR | PCDH12 |
| SORBS2 | C5orf45 |
| TDO2 | SEMA5A |
| ZNF718 | CMYA5 |
| BMP2K | CCDC125 |
| MFSD10 | SPEF2 |
| WFS1 | OR2V1 |
| FAT1 | ADAM19 |
| ASIC5 | C5orf38 |
| GALNTL6 | KIAA1024L |
| RNF212 | GZMK |
| TMEM156 | FGFR4 |
| FAM160A1 | AQPEP |
| ZFYVE28 | APC |
| MAML3 | MCC |
| POU4F2 | NMUR2 |
| COMMD8 | DIAPH1 |
| DSPP | KCNN2 |
| DCHS2 | SLU7 |
| EVC | APBB3 |
| HTT | SOWAHA |
| SDHA | WWC1 |
| SEC24A | DNAH5 |
| MAP3K1 | C5orf58 |
| ZDHHC11 | PRDM9 |
| ARHGEF28 | PARP8 |
| TRIM23 | FAM105A |
| LRRC70 | ECI2 |
| PCDHGB4 | BCLAF1 |
| C5orf60 | MRPL2 |
| ADAMTS6 | LCA5 |
| PCDHA7 | PACSIN1 |
| SYNPO | USP49 |
| MYOT | DST |
| PCDHA4 | TBC1D7 |
| SPEF2 | NHSL1 |
| BTNL8 | ENPP1 |
| SLIT3 | SLC22A23 |
| NOP16 | GPR63 |
| SLC9A3 | FBXO30 |
| KIAA1024L | TAAR8 |
| RPL26L1 | DSP |
| AQPEP | EZR |
| IL9 | TAGAP |
| SLC26A2 | FANCE |
| C9 | FAM120B |
| SLU7 | SNAP91 |
| GZMK | NUP153 |
| AP3B1 | ARMC12 |
| GPRIN1 | IGF2R |
| C5orf45 | TAAR5 |
| GDF9 | RNASET2 |
| ADAM19 | HLA-DRB5 |
| CCDC125 | CTGF |
| PCDH12 | OOEP |
| BRD9 | HLA-B |
| CMYA5 | WDR27 |
| FBXL7 | TFB1M |
| C5orf58 | CDC40 |
| MCC | MAP3K4 |
| OR2V1 | SYNE1 |
| DNAH5 | TIAM2 |
| PCDHA9 | HLA-DQB1 |
| AHRR | C6orf223 |
| PCDH1 | MTRF1L |
| FGFR4 | TDRD6 |
| APBB3 | ADGB |
| PRDM9 | ATXN1 |
| NMUR2 | PPP1R3G |
| SOWAHA | TBP |
| PARP8 | C6orf1 |
| IQGAP2 | SPACA1 |
| DIAPH1 | GTF3C6 |
| FAM105A | PPP1R14C |
| DHX29 | NCOA7 |
| KCNN2 | UTRN |
| PCDHB10 | HLA-DRB1 |
| WWC1 | HLA-DRB1 |
| BCLAF1 | DAAM2 |
| SNAP91 | TARP |
| ECI2 | OR2A1, OR2A42 |
| DST | KMT2C |
| MRPL2 | PSPH |
| TFB1M | EGFR |
| PACSIN1 | COBL |
| GPR63 | MUC3A |
| SYNE1 | MUC17 |
| RNASET2 | SSC4D |
| TAAR8 | METTL2B |
| FBXO30 | AOAH |
| DSP | PRSS1 |
| SLC22A23 | ZNF479 |
| ENPP1 | MACC1 |
| TBC1D7 | DNAH11 |
| FAM120B | POM121 |
| NUP153 | IQCE |
| ARMC12 | C7orf25 |
| EZR | MAGI2 |
| IGF2R | POM121C |
| USP49 | CTAGE4 |
| OOEP | STEAP1B |
| HLA-B | SEMA3D |
| HLA-DQB1 | PIK3CG |
| FANCE | MGAM |
| TAGAP | PKD1L1 |
| MAP3K4 | KIAA1549 |
| CTGF | STK31 |
| TDRD6 | MEOX2 |
| HLA-DRB5 | WBSCR27 |
| TIAM2 | PCLO |
| ADGB | GPER1 |
| TAAR5 | POR |
| MTRF1L | SLC26A5 |
| CDC40 | CTTNBP2 |
| TBP | ZNF789 |
| PPP1R14C | DNAJC30 |
| UTRN | RSPH10B, RSPH10B2 |
| WDR27 | FOXP2 |
| SPACA1 | SRRM3 |
| GTF3C6 | CFTR |
| HLA-DRB1 | MUC12 |
| PPP1R3G | ZNF273 |
| CNPY3 | CLCN1 |
| ATXN1 | CASP2 |
| C6orf1 | PODXL |
| NCOA7 | GET4 |
| C6orf223 | AKAP9 |
| DAAM2 | NOBOX |
| OR2A1, OR2A42 | LFNG |
| EGFR | GPR141 |
| COBL | SSPO |
| KMT2C | IRF5 |
| METTL2B | PMS2 |
| STK31 | KLF14 |
| MUC3A | AP5Z1 |
| PSPH | WIPI2 |
| ZNF479 | CTAGE15 |
| SSC4D | TMEM184A |
| TARP | FAM115C |
| MUC17 | EPPK1 |
| SEMA3D | CSMD3 |
| AOAH | MAL2 |
| PRSS1 | DDHD2 |
| CTAGE4 | PLEC |
| ZNF789 | ANXA13 |
| PKD1L1 | SDR16C5 |
| POM121 | GPT |
| C7orf25 | RIMS2 |
| FOXP2 | PCMTD1 |
| WBSCR27 | PLAT |
| DNAH11 | RP1L1 |
| STEAP1B | NSMCE2 |
| POM121C | CHD7 |
| GPER1 | MFSD3 |
| CFTR | CNGB3 |
| ZNF273 | UNC5D |
| CLCN1 | INTS10 |
| MAGI2 | SCRIB |
| IQCE | TNKS |
| C7orf60 | ZNF7 |
| KIAA1549 | SGK223 |
| LFNG | CYP11B1 |
| MGAM | FDFT1 |
| PCLO | MAFA |
| SRRM3 | FGL1 |
| SLC26A5 | PRSS3 |
| MEOX2 | FAM205A |
| MUC12 | VPS13A |
| DNAJC30 | CACNA1B |
| AKAP9 | ABCA1 |
| CASP2 | MAMDC4 |
| VSTM2A | NDOR1 |
| NOBOX | 1-Dec |
| PODXL | OR13C5 |
| NCF1 | ORM1 |
| GPR141 | ABCA2 |
| SSPO | AQP7 |
| GET4 | CCBL1 |
| WIPI2 | ASPN |
| IRF5 | RABGAP1 |
| CTAGE15 | OR1Q1 |
| FAM115C | NUTM2F |
| PMS2 | DENND4C |
| AP5Z1 | ENTPD8 |
| KLF14 | FANCC |
| MAL2 | CNTNAP3B |
| SQLE | NUTM2G |
| CHD7 | FUT7 |
| DDHD2 | LURAP1L |
| RIMS2 | PCSK5 |
| SDR16C5 | IFNA10 |
| GPT | NOL8 |
| TNFRSF11B | AK8 |
| EPPK1 | ADAMTS13 |
| PLEC | EPB41L4B |
| RP1L1 | FAM157B |
| PCMTD1 | WDR34 |
| ANXA13 | SPATA31C2 |
| TNKS | BRWD3 |
| PLAT | TBX22 |
| GGH | TGIF2LX |
| NSMCE2 | P2RY4 |
| FDFT1 | MAGIX |
| OSGIN2 | ZMAT1 |
| CSMD1 | OR13H1 |
| MFSD3 | TEX11 |
| ZNF7 | RBMXL3 |
| FER1L6 | MAGEA10 |
| FGL1 | TAF7L |
| SGK223 | SSX5 |
| SCRIB | P2RY10 |
| ZFHX4 | MAGEA1 |
| VPS13A | SLC25A43 |
| PRSS3 | POU3F4 |
| ABCA1 | ARMCX4 |
| OR13C2 | CDK16 |
| DEC1 | GPR112 |
| FAM205A | KDM6A |
| CACNA1B | MAGEC1 |
| OR13C5 | GLRA4 |
| MAMDC4 | GUCY2F |
| FANCC | SYTL4 |
| AQP7 | IRS4 |
| NDOR1 | NUDT11 |
| SEC16A | MAGEE2 |
| FPGS | FRMD7 |
| ORM1 | RPGR |
| ASPN | BCORL1 |
| RABGAP1 | TCEAL6 |
| DENND4C | POF1B |
| CCBL1 | MXRA5 |
| LURAP1L | GABRQ |
| NOL8 | TBC1D25 |
| ABCA2 | EGFL6 |
| RGS3 | WDR13 |
| OR1Q1 | ZCCHC16 |
| ENTPD8 | VBP1 |
| IFNA10 | MTMR8 |
| PCSK5 | TAB3 |
| NUTM2F | FOXR2 |
| CNTNAP3B | NHS |
| FAM157B | SLC25A5 |
| AK8 | EDA2R |
| NUTM2G | ATXN3L |
| ADAMTS13 | GPC3 |
| FUT7 | RBMX |
| EPB41L4B | ARSD |
| FOXE1 | UBE2NL |
| HRCT1 | CTAG2 |
| BRWD3 | DCAF8L2 |
| MAGEC1 | TEX13A |
| GPR112 | MAGEC3 |
| MAGEA10 | SUPT20HL1 |
| TEX11 | SHROOM2 |
| OR13H1 | PLXNB3 |
| MAGIX | AMER1 |
| MAGEA1 | LRCH2 |
| TGIF2LX | KAL1 |
| ZMAT1 | F8 |
| EGFL6 | DMD |
| SLC25A43 | PNMA3 |
| POF1B | FAM120C |
| CSAG1 | MAGEB10 |
| VBP1 | ATP7A |
| UBE2NL | HDHD1 |
| MXRA5 | CXorf40A |
| F8 | MAGEB16 |
| RBMXL3 | PRRG3 |
| RPGR | FLJ44635 |
| CTAG2 | GRIA3 |
| GUCY2F | BCOR |
| GABRQ | MAGEB18 |
| DMD | TMEM187 |
| SSX5 | GABRE |
| RBMX | RGAG4 |
| IRS4 | CSAG1 |
| CDK16 | HEPH |
| KDM6A | XG |
| ARSD | MAP3K15 |
| P2RY4 | PAGE2 |
| MTMR8 | RAI2 |
| ATXN3L | HCFC1 |
| TAB3 | GYG2 |
| GLRA4 | OPN1LW |
| MAGEB16 | SLC7A3 |
| MAGEC3 | MAGEB3 |
| DCAF8L2 | KIAA1210 |
| SYTL4 | ARSE |
| P2RY10 | CXorf30 |
| WDR13 | ATP11C |
| POU3F4 | IL1RAPL1 |
| SLC25A5 | FAM127B |
| TCEAL6 | MAGEB2 |
| HDHD1 | SLC16A2 |
| MAGEB18 | VCX2 |
| GYG2 | VCX |
| NUDT11 | CD24 |
| SUPT20HL1 | PCDH11Y |
| ZCCHC16 | |
| TAF7L |  |
| TEX13A |  |
| BCORL1 |  |
| FRMD7 |  |
| MAGEE2 |  |
| PRRG3 |  |
| CXorf30 |  |
| ARMCX4 |  |
| TBC1D25 | |
| SHROOM2 | |
| RGAG4 |  |
| NHS |  |
| FOXR2 |  |
| EDA2R |  |
| GPC3 |  |
| ATP7A |  |
| TMEM187 | |
| PLXNB3 |  |
| PNMA3 |  |
| GRIA3 |  |
| PAGE2 |  |
| MAGEB10 | |
| CXorf40A | |
| FLJ44635 |  |
| KAL1 |  |
| MAP3K15 | |
| FAM120C | |
| AMER1 |  |
| GABRE |  |
| RAI2 |  |
| ATP11C |  |
| MAGEB3 |  |
| HEPH |  |
| FAM127B | |
| OPN1LW |  |
| XG |  |
| BCOR |  |
| ARSE |  |
| MAGEB2 |  |
| KIAA1210 | |
| VCX2 |  |
| ARHGAP4 | |
| SLC7A3 |  |
| PCDH11Y | |
| CD24 |  |
