# Supplementary material for: Characterization of genetic alterations in brain metastases from non‐small cell lung cancer
Source: FEBS Open Bio. 2018 Aug 30;8(9):1544–52. doi: 10.1002/2211-5463.12501 (PMC6120240; doi:10.1002/2211-5463.12501)
Supplement: Supplementary file 2 — Table S2. Mutant genes identified in P2 primary tumor and matched brain metastasis samples. [file FEB4-8-1544-s002.docx]

**Supplemental table 2. Mutant genes identified in P2 primary tumors and matched brain metastases samples.**

| **P2- primary tumors** | **P2- brain metastases** |
| --- | --- |
| FLG | PDE4DIP |
| PRAMEF2 | MST1L |
| CEP170 | DPYD |
| FMO1 | NOTCH2NL |
| PDE4DIP | GPATCH2 |
| HEYL | RPTN |
| OR2T35 | HNRNPCL1, HNRNPCL3, HNRNPCL4 |
| NOTCH2NL | OR2T3 |
| DPYD | FH |
| HRNR | FCGR3A |
| GPATCH2 | OR2T34 |
| OR10J1 | FLG |
| FCGR3A | OR2T35 |
| TNR | MAST2 |
| OR2T3 | SCAMP3 |
| MAST2 | HRNR |
| TCHH | CDK11A, CDK11B |
| SMYD3 | OR10J1 |
| OR2L2 | OR2L2 |
| WASF2 | OR2T2 |
| OR6N2 | POGK |
| HNRNPCL1, HNRNPCL3, HNRNPCL4 | TCHH |
| PPIAL4G | PPIAL4G |
| SCAMP3 | SMYD3 |
| MST1L | TNR |
| FH | OR2T4 |
| CEP350 | HNRNPCL2 |
| POGK | OR2L3 |
| SUCO | LSM10 |
| FLG2 | ENAH |
| CCDC18 | PRAMEF2 |
| GON4L | KIF2C |
| OR2T2 | GPSM2 |
| HNRNPCL2 | WASF2 |
| WDR64 | LCE4A |
| OR2L8 | OBSCN |
| ASPM | FLG2 |
| LSM10 | OR2L8 |
| DISP1 | OR2T27 |
| CRB1 | CRB1 |
| EPB41 | EPS8L3 |
| OR2T34 | GON4L |
| LPHN2 | KIAA1804 |
| NBPF8 | OR2T5 |
| SLC44A3 | OR6N2 |
| ITLN2 | DENND4B |
| USH2A | PER3 |
| PRAMEF1 | WDR64 |
| HSPA6 | C1orf210 |
| KIF2C | CEP350 |
| KIAA1804 | SUCO |
| OR2L3 | DISP1 |
| OR2T5 | BMP8B |
| OR4F5 | LRRC38 |
| AP4B1 | EPB41 |
| NOTCH2 | ASPM |
| OBSCN | COL16A1 |
| OR2T4 | PRAMEF1 |
| OR2T27 | NBPF8 |
| USP48 | EPHA8 |
| METTL11B | DMBX1 |
| EPS8L3 | ITLN2 |
| C1orf210 | CCDC18 |
| IGFN1 | USH2A |
| NBPF10 | RGS16 |
| EPHA8 | HSPA6 |
| OR6P1 | OR4F5 |
| CDK11A, CDK11B | METTL11B |
| KCNA10 | NOTCH2 |
| BRDT | CTNNBIP1 |
| PPAP2B | AP4B1 |
| BMP8B | SLC44A3 |
| LRRC38 | AXDND1 |
| OR2T1 | OR2T12 |
| RGS16 | LAMB3 |
| CFHR1 | CDCP2 |
| CD2 | KCNA10 |
| OR2T12 | USP48 |
| C1orf94 | OXCT2 |
| NBPF15 | ARHGEF10L |
| LOC101929983, PRAMEF6, PRAMEF9 | OR2T1 |
| OXCT2 | OR6P1 |
| MR1 | NBPF10 |
| ARHGEF10L | PPAP2B |
| LAMB3 | MR1 |
| DENND1B | TMEM52 |
| NIT1 | USP1 |
| CTNNBIP1 | OR2T29 |
| FOXJ3 | FCAMR |
| H3F3A | MOV10 |
| MAGI3 | CFHR2 |
| OR2T29 | LAD1 |
| LOC391003, PRAMEF22 | HEYL |
| EFCAB14 | EFCAB14 |
| OR2T8 | SPRR3 |
| NBPF14, NBPF8, NBPF9 | CD2 |
| BMP8A | BMP8A |
| CSF3R | OR2T8 |
| HIVEP3 | FOXJ3 |
| DCST2 | CDK11B |
| RAB3GAP2 | LOC101929983, PRAMEF6, PRAMEF9 |
| CDK11B | CSF3R |
| TOMM40L | CFHR1 |
| LEFTY1 | ESPN |
| ESPN | IGFN1 |
| ASAP3 | LOC391003, PRAMEF22 |
| CROCC | SLC35E2 |
| TMEM52 | NBPF15 |
| SLC35E2B | AIM1L |
| AIM1L | NBPF14, NBPF8, NBPF9 |
| AKR7A3 | BRDT |
| KIAA1522 | KCNN3 |
| LCE4A | PRAMEF4 |
| ENAH | NIT1 |
| DMBX1 | DCST2 |
| KCNN3 | MIR205HG |
| KIAA0040 | MAGI3 |
| RPTN | SLC25A33 |
| GPSM2 | HIVEP3 |
| AXDND1 | PLEKHG5 |
| DENND4B | ASAP3 |
| PER3 | KIAA1522 |
| USP1 | MXRA8 |
| SPRR3 | LEFTY1 |
| MIR205HG | AKR7A3 |
| CDCP2 | DENND1B |
| RSG1 | TOMM40L |
| CFHR2 | CROCC |
| FCAMR | TNFRSF18 |
| LAD1 | STRIP1 |
| FAM208B | ACOT7 |
| LOC100996758, NPY4R | UBXN11 |
| SYT15 | SYT15 |
| LYZL2 | LOC100996758, NPY4R |
| WAC | BICC1 |
| RPP38 | GPRIN2 |
| BICC1 | FAM208B |
| ZNF239 | WAC |
| GAD2 | KIN |
| GPRIN2 | LYZL2 |
| FAM160B1 | FAM160B1 |
| FZD8 | RBM20 |
| ZNF37A | EIF5AL1 |
| TIMM23 | FRG2B |
| EIF5AL1 | TIMM23 |
| SLC16A9 | RPP38 |
| ASCC1 | FZD8 |
| ADARB2 | ZNF239 |
| RBM20 | AFAP1L2 |
| FRG2B | SLC16A9 |
| JMJD1C | GAD2 |
| ANKRD30A | COL17A1 |
| NEBL | AGAP9 |
| COL17A1 | RRP12 |
| RET | ECHDC3 |
| AGAP9 | JMJD1C |
| ECHDC3 | LIPN |
| LIPN | ZNF37A |
| MKI67 | MKI67 |
| PNLIPRP1 | AGAP5 |
| GSTO1 | GSTO1 |
| RBP3 | PNLIPRP1 |
| LRRC27 | KAZALD1 |
| MCU | LRRC27 |
| AGAP5 | NEBL |
| TACC2 | RET |
| CHST3 | TACC2 |
| RRP12 | CHST3 |
| AFAP1L2 | CHUK |
| KIN | BBOX1 |
| OR4C3 | OR8U1, OR8U8 |
| MUC6 | OR4C3 |
| BBOX1 | SAA2, SAA2-SAA4 |
| OR8U1, OR8U8 | OR9G1, OR9G9 |
| OR9G1, OR9G9 | OR8B2 |
| OR8B2 | OR8G2 |
| OR8B3 | OR8B3 |
| TRIM49C | MUC6 |
| OR8G2 | SERPING1 |
| LRRC4C | MUC5B |
| SAA2, SAA2-SAA4 | DSCAML1 |
| SERPING1 | TRIM49C |
| MMP20 | CCDC87 |
| CCDC87 | MAPK8IP1 |
| NCAPD3 | OR51G1 |
| OR51G1 | GPR137 |
| OR8U1, OR8U8 | OR52R1 |
| OR52R1 | LRRC4C |
| DSCAML1 | ABCC8 |
| CDON | DCHS1 |
| MAPK8IP1 | OR8U1, OR8U8 |
| MUC5B | CDON |
| NLRP14 | CDHR5 |
| ALKBH8 | NLRP14 |
| GPR137 | NCAPD3 |
| DCHS1 | MYEOV |
| NEU3 | C11orf40 |
| BAD | MMP20 |
| RRM1 | ALKBH8 |
| OR1S1 | SAAL1 |
| KCNJ5 | NEU3 |
| CDHR5 | BAD |
| OR10G4 | TMPRSS13 |
| OR8U1 | SCGB1C1, SCGB1C2 |
| ABCC8 | KCNJ5 |
| MUC2 | OR10G4 |
| SCGB1C1, SCGB1C2 | MUC2 |
| OR4C12 | TMEM25 |
| NRIP3 | NRIP3 |
| TMEM25 | OR1S1 |
| USP35 | OR4C12 |
| B4GALNT4 | FAM86C1 |
| IGHMBP2 | USP35 |
| KRTAP5-7 | KRTAP5-7 |
| CARNS1 | B4GALNT4 |
| SAAL1 | ASRGL1 |
| MICALCL | IFITM3 |
| TMPRSS13 | MICALCL |
| TMEM223 | IGHMBP2 |
| C11orf40 | ESRRA |
| MYEOV | TMEM223 |
| CHRDL2 | C11orf80 |
| TAS2R19 | LTBP3 |
| GUCY2C | CARNS1 |
| TMEM116 | SCUBE2 |
| PRB2 | SAA1 |
| PHC1 | KRTAP5-5 |
| TAS2R46 | TMEM151A |
| TAS2R30 | DGKZ |
| TAS2R43 | TAS2R19 |
| KLRF2 | PHC1 |
| USP5 | TAS2R43 |
| TAS2R31 | TAS2R46 |
| KRT6A | TAS2R31 |
| GAS2L3 | TAS2R30 |
| KLRC2 | KRT6A |
| FGD6 | USP5 |
| KRT6B | GAS2L3 |
| KMT2D | FGD6 |
| SCAF11 | OR10A7 |
| PIK3C2G | KRT6B |
| CD163L1 | KLRC2 |
| SLC2A3 | KMT2D |
| PLCZ1 | DCP1B |
| SLC15A5 | SCAF11 |
| COL2A1 | KLRF2 |
| FAM186A | CD63 |
| WBP11 | FAM216A |
| C12orf4 | KRT3 |
| SP1 | CD163L1 |
| CD63 | CELA1 |
| OR6C4 | APOF |
| CCDC184 | WIBG |
| PRB1 | FAM186A |
| EP400 | SLC2A3 |
| NUP107 | OR6C4 |
| DNAH10 | DUSP16 |
| FOXN4 | PRB1 |
| KNTC1 | SP1 |
| FIGNL2 | KRT2 |
| TWF1 | SLC15A5 |
| ESYT1 | WBP11 |
| DCP1B | CHFR |
| KRT2 | ZNF384 |
| CAMKK2 | NTS |
| WDR66 | CCDC184 |
| KRT3 | EP400 |
| DUSP16 | PHLDA1 |
| ZNF384 | RASAL1 |
| PLBD1 | PLCZ1 |
| PHLDA1 | FOXN4 |
| PEX5 | PRB2 |
| CELA1 | PLBD1 |
| PABPC3 | FIGNL2 |
| SKA3 | C12orf4 |
| TPTE2 | CAMKK2 |
| CKAP2 | WDR66 |
| FLT1 | ESYT1 |
| PSPC1 | CNOT2 |
| KLF5 | KNTC1 |
| C1QTNF9 | DNAH10 |
| EXOSC8 | NUP107 |
| PARP4 | OS9 |
| ZMYM5 | PRB3 |
| SLITRK5 | KRT18 |
| FLT3 | BRI3BP |
| RNF6 | PLBD2 |
| SETDB2 | PABPC3 |
| WDR89 | CKAP2 |
| MLH3 | PCID2 |
| OR4K1 | PSPC1 |
| GPR65 | SKA3 |
| SEL1L | IL17D |
| FAM181A | CCDC168 |
| RPGRIP1 | SLITRK5 |
| PLEKHG3 | TPTE2 |
| SIPA1L1 | FREM2 |
| POTEG | C1QTNF9 |
| DCAF11 | LRCH1 |
| AHNAK2 | KLF5 |
| HOMEZ | PARP4 |
| OR4N2 | EXOSC8 |
| POTEM | ZMYM5 |
| ATG2B | RNF6 |
| ADAM21 | DACH1 |
| EXOC3L4 | GPR180 |
| RBM23 | FLT3 |
| DCAF5 | RBM23 |
| REC8 | FAM181A |
| RIN3 | OR4K1 |
| TMEM121 | GPR65 |
| MPI | DCAF5 |
| UNC13C | AHNAK2 |
| OR4M2 | MLH3 |
| FANCI | RIN3 |
| LDHAL6B | POTEG |
| WDR72 | RPGRIP1 |
| FAM154B | SEL1L |
| TYRO3 | PLEKHG3 |
| EIF2AK4 | REC8 |
| CYP1A2 | BDKRB2 |
| ATP8B4 | HOMEZ |
| GOLGA8R | SYT16 |
| ADAMTS17 | SIPA1L1 |
| CASC5 | POTEM |
| VIMP | ATG2B |
| CATSPER2 | OR4N2 |
| ATP10A | TMEM121 |
| NUTM1 | EXOC3L4 |
| MAN2C1 | ADAM21 |
| ASB7 | IRF2BPL |
| GCNT3 | ACOT2 |
| HCN4 | MAP3K9 |
| PHGR1 | OR4M2 |
| MEF2A | UNC13C |
| CD276 | TYRO3 |
| OTOA | GOLGA8R |
| RRN3 | WDR72 |
| CES1 | FANCI |
| ATXN1L | FAM154B |
| GOT2 | CYP1A2 |
| SRCAP | ADAMTS17 |
| SULT1A1 | EIF2AK4 |
| TNRC6A | MEF2A |
| E4F1 | ATP8B4 |
| PDPR | VIMP |
| ZFHX3 | MAN2C1 |
| NUPR1 | CASC5 |
| FAM57B | CATSPER2 |
| GTF3C1 | NUTM1 |
| FBRS | CD276 |
| NOB1 | ASB7 |
| CLEC18B | ATP10A |
| GLYR1 | HCN4 |
| PHLPP2 | MPI |
| CORO7, CORO7-PAM16 | GCNT3 |
| ZNF469 | GOLGA8A |
| C16orf95 | LOC283710 |
| IRX6 | FSD2 |
| MYH11 | GOLGA6L1, GOLGA6L22 |
| RPUSD1 | CHRNA3 |
| IFT140 | GOLGA8M |
| TMEM204 | HERC2 |
| TPSD1 | RRN3 |
| FLYWCH1 | CES1 |
| TPSAB1 | SRCAP |
| PKD1 | C16orf93 |
| IL4R | ZNF423 |
| IL27 | E4F1 |
| ZFPM1 | PDPR |
| MTSS1L | E2F4 |
| TPSB2 | GOT2 |
| LOC100129697 | OTOA |
| ABCA3 | SULT1A1 |
| DECR2 | CORO7, CORO7-PAM16 |
| E2F4 | CLEC18B |
| C16orf93 | FBRS |
| OGFOD1 | FAM57B |
| PKD1L2 | NOB1 |
| MAP2K3 | NOMO1 |
| NCOR1 | ZNF469 |
| TVP23C, TVP23C-CDRT4 | ZFHX3 |
| KCNJ12, KCNJ18 | PKD1L2 |
| EPN3 | OGFOD1 |
| FBXW10 | DHX38 |
| SLFN14 | GTF3C1 |
| KIAA1731NL | PKD1 |
| DNAH17 | RPUSD1 |
| USP32 | TMEM204 |
| ANKRD40 | GLYR1 |
| SMG6 | TLDC1 |
| NFE2L1 | NUPR1 |
| FASN | TPSAB1 |
| AXIN2 | IFT140 |
| TAOK1 | TPSD1 |
| GALK1 | IL4R |
| FBXO39 | C16orf95 |
| SPATA20 | FLYWCH1 |
| TP53 | IRX6 |
| CCDC57 | ZFPM1 |
| USP6 | ABCA3 |
| ENTHD2 | MYH11 |
| TRIM16 | IL27 |
| OR1E1 | MTSS1L |
| CDC27 | JPH3 |
| ZNF750 | TPSB2 |
| KIF18B | PHLPP2 |
| B3GNTL1 | DECR2 |
| C17orf104 | GFER |
| DNAH9 | CHTF18 |
| SLFN13 | APOBR |
| YBX2 | GSPT1 |
| PLEKHH3 | UNKL |
| ABR | LOC100129697 |
| GSDMA | ZKSCAN2 |
| TBC1D28 | KCNJ12, KCNJ18 |
| OR1D5 | MAP2K3 |
| LGALS9C | CALCOCO2 |
| FADS6 | EPN3 |
| TAF15 | KIAA1731NL |
| KDM6B | ANKRD40 |
| QRICH2 | SARM1 |
| KRTAP4-1 | DNAH17 |
| MPRIP | FBXW10 |
| USP36 | SLFN14 |
| SARM1 | SMG6 |
| RAI1 | FBXO39 |
| ATP9B | AXIN2 |
| ZADH2 | NFE2L1 |
| CCDC102B | GALK1 |
| POTEC | FASN |
| SMCHD1 | KRTAP9-4 |
| CNDP1 | TAOK1 |
| SLC35G4 | SPATA20 |
| MEX3C | P4HB |
| ZNF440 | USP6 |
| RFPL4A | DNAH9 |
| ZNF14 | QRICH2 |
| RFPL4AL1 | TRIM16 |
| FCGBP | ENTHD2 |
| ZNF799 | TEX14 |
| PDE4C | TP53 |
| HAUS5 | USP32 |
| ZNF208 | CCDC57 |
| FFAR3 | FADS6 |
| ZNF536 | EVPL |
| MUC16 | B3GNTL1 |
| SPTBN4 | MPRIP |
| ZNF841 | KIF18B |
| SPHK2 | RAI1 |
| RSPH6A | OR1E1 |
| PLIN4 | ZNF750 |
| ILF3 | SCARF1 |
| LILRA1 | MYO15A |
| TICAM1 | SMYD4 |
| GPR32 | KRTAP4-1 |
| OR7A5 | GSDMA |
| AKAP8L | SLFN13 |
| DMRTC2 | ABR |
| RBM42 | NCOR1 |
| SRRM5 | KRTAP9-2 |
| FCER2 | CDC27 |
| CLASRP | PLEKHH3 |
| BBC3 | KRTAP9-8 |
| LILRA6, LILRB3 | C17orf104 |
| LILRB1 | USP36 |
| ANKLE1 | KDM6B |
| FFAR1 | KRTAP9-9 |
| PTGER1 | TBC1D28 |
| C19orf26 | OR1D5 |
| OR7E24 | GSG2 |
| SIGLEC11 | CBX4 |
| ZNF814 | KRT10 |
| CPAMD8 | LGALS9C |
| ZNF578 | ATP9B |
| NACC1 | POTEC |
| SIPA1L3 | ZADH2 |
| LSR | SMCHD1 |
| HSPBP1 | CCDC102B |
| URI1 | CNDP1 |
| ZNF83 | MEX3C |
| ZNF544 | ANKRD30B |
| DNAH7 | FCGBP |
| ANKRD36 | RFPL4A |
| CNPPD1 | URI1 |
| LTBP1 | FFAR3 |
| CARF | LILRA1 |
| CPS1 | MUC16 |
| MTIF2 | PDE4C |
| XDH | LILRA2 |
| EML6 | ZNF536 |
| TUBA3E | ZNF257 |
| NEB | ZNF14 |
| XIRP2 | RFPL4AL1 |
| MSH2 | ZNF440 |
| GPR1 | AKAP8L |
| DYSF | TICAM1 |
| CDKL4 | LILRB3 |
| PXDN | ZNF799 |
| ARHGAP25 | HAUS5 |
| USP37 | NDUFS7 |
| BAZ2B | PLIN4 |
| CYP27A1 | LILRB2 |
| TTN | SPHK2 |
| NCAPH | SIGLEC11 |
| WNT10A | LSR |
| CCL20 | ZNF208 |
| RAD51AP2 | RSPH6A |
| HECW2 | LILRB1 |
| OTOF | C19orf73 |
| EIF2AK2 | SPTBN4 |
| ZFP36L2 | ZNF841 |
| FAM110C | FCER2 |
| REG3A | OR7A5 |
| GCKR | ZNF615 |
| POTEE | FFAR1 |
| OR6B3 | EPN1 |
| OXER1 | CLASRP |
| TCF23 | LILRA6, LILRB3 |
| POTEF | SRRM5 |
| HS6ST1 | BBC3 |
| FBXO41 | EMR2 |
| HOXD9 | SIPA1L3 |
| C2orf81 | DMRTC2 |
| STK17B | C19orf26 |
| SPATA3 | SMARCA4 |
| KIF1A | PTGER1 |
| GIGYF2 | ZIK1 |
| PHACTR3 | ZNF83 |
| CASS4 | ZNF578 |
| KIF16B | OR7E24 |
| RPRD1B | ZNF544 |
| BPIFB4 | ANKLE1 |
| NPBWR2 | C19orf52 |
| SLC2A10 | SSC5D |
| TRPC4AP | CPAMD8 |
| MACROD2 | LILRA3 |
| ATP9A | HSPBP1 |
| CNBD2 | NACC1 |
| ADIG | CYP4F2 |
| NINL | ZNF787 |
| NPEPL1 | DMKN |
| BPIFB3 | UPK1A |
| SIRPB1 | NR2F6 |
| RALY | PCSK4 |
| NCOA3 | CNN2 |
| DEFB132 | LTBP1 |
| DEFB126 | DYSF |
| SLC23A2 | TUBA3E |
| TPTE | SPATA3 |
| IFNAR2 | STK17B |
| BAGE2, BAGE3 | MTIF2 |
| DOPEY2 | CARF |
| C21orf140 | ANKRD36 |
| ETS2 | XIRP2 |
| URB1 | GPR1 |
| KRTAP10-7 | CPS1 |
| ADAMTS1 | PXDN |
| KRTAP10-2 | ARHGAP25 |
| FTCD | EML6 |
| SH3BGR | NEB |
| KRTAP10-1 | DNAH7 |
| COL18A1 | FAM110C |
| CYB5R3 | CYP27A1 |
| SPECC1L | CDKL4 |
| ISX | ZFP36L2 |
| TCN2 | MSH2 |
| EP300 | TMEM247 |
| CSDC2 | OTOF |
| MORC2 | KIF1A |
| ZNF70 | WNT10A |
| L3MBTL2 | CCL20 |
| CLTCL1 | CNPPD1 |
| CABIN1 | USP37 |
| KIAA0930 | REG3A |
| SUSD2 | HOXD9 |
| TBX1 | GCKR |
| GUCD1 | ALPPL2 |
| TRIOBP | ERBB4 |
| CPT1B | BAZ2B |
| NEFH | CGREF1 |
| CDC42EP1 | RAB6C |
| SRRD | TTN |
| MIEF1 | OXER1 |
| MUC20 | CCDC74A |
| TSC22D2 | NCAPH |
| PPP2R3A | OR6B3 |
| MAGI1 | C2orf81 |
| SETD2 | TCF23 |
| ZNF717 | HS6ST1 |
| FANCD2 | EIF2AK2 |
| TRANK1 | GIGYF2 |
| ZBBX | FBXO41 |
| BAP1 | POTEF |
| COLQ | ALMS1 |
| CX3CR1 | PHACTR3 |
| HIGD1A | CASS4 |
| EDEM1 | DEFB126 |
| KIF9 | KIF16B |
| PDZRN3 | NCOA3 |
| MST1 | BPIFB4 |
| DLEC1 | RPRD1B |
| SNRK | SLC2A10 |
| MUC4 | NPBWR2 |
| MYH15 | NINL |
| TRAK1 | FASTKD5 |
| CCDC66 | DEFB132 |
| MAGEF1 | TRPC4AP |
| KIAA2018 | ATP9A |
| NCKIPSD | MACROD2 |
| TMIE | CNBD2 |
| FAM157A | ADIG |
| ATG3 | NPEPL1 |
| LRRC66 | MKKS |
| FRG1 | BPIFB3 |
| WDR19 | RALY |
| HERC5 | SIRPB1 |
| OTOP1 | CEP250 |
| FAT1 | BAGE2, BAGE3 |
| ANK2 | TPTE |
| RGS12 | DOPEY2 |
| ZNF141 | KRTAP10-1 |
| MRPS18C | ETS2 |
| TRIML1 | C21orf140 |
| PF4 | URB1 |
| RNF212 | KRTAP10-7 |
| UVSSA | KRTAP10-2 |
| DSPP | FTCD |
| POU4F2 | ADAMTS1 |
| ALPK1 | COL18A1 |
| FHDC1 | SH3BGR |
| MAML3 | IFNAR2 |
| WHSC1 | ABCG1 |
| DCHS2 | KRTAP10-9 |
| ZDHHC11 | BAGE, BAGE2, BAGE3, BAGE4, BAGE5 |
| GRXCR2 | KRTAP10-6 |
| RELL2 | CYB5R3 |
| BRD9 | ISX |
| PCDHGA2 | SPECC1L |
| SLC6A7 | EP300 |
| PCDHGA4 | TCN2 |
| SPEF2 | NEFH |
| C5orf42 | CSDC2 |
| ADAM19 | MIEF1 |
| PCDHA6 | ZNF70 |
| NSD1 | KIAA0930 |
| C5orf58 | CABIN1 |
| MYOT | MORC2 |
| KIAA0825 | CLTCL1 |
| GEMIN5 | L3MBTL2 |
| SLC26A2 | CPT1B |
| BDP1 | GUCD1 |
| NIPBL | SUSD2 |
| PCDHA9 | TRIOBP |
| C5orf45 | TBX1 |
| SLU7 | CDC42EP1 |
| ZNF454 | CYP2D6 |
| HMGCR | BCR |
| AP3B1 | MUC20 |
| FAM105A | COPG1 |
| TCF7 | GPR149 |
| PCDHGA8 | PPP2R3A |
| PCDHB13 | MAGI1 |
| TRIM7 | TOPBP1 |
| PRDM9 | PARP15 |
| FAM153B | ZNF717 |
| PCDHB2 | SETD2 |
| PCDHB10 | PLD1 |
| SOWAHA | TRANK1 |
| MCC | ATG3 |
| KCNN2 | LNP1 |
| PPP2R2B | FANCD2 |
| MAP3K1 | PIGX |
| WWC1 | C3orf80 |
| JMY | COLQ |
| ADAMTS2 | TRAK1 |
| NOP16 | ZBBX |
| SDHA | ROBO1 |
| IK | PLXND1 |
| GPRIN1 | USP13 |
| SYNE1 | EDEM1 |
| RHAG | CEP63 |
| TCP10L2 | CX3CR1 |
| BCLAF1 | MAGEF1 |
| TCP10 | KIAA2018 |
| GPR126 | KIF9 |
| AHI1 | PDZRN3 |
| FBXO30 | HIGD1A |
| TAAR6 | RBM5 |
| MDN1 | MAATS1 |
| KCNK16 | MST1 |
| SHPRH | SLC22A13 |
| HUS1B | TMIE |
| TCF21 | DLEC1 |
| LAMA2 | CCDC66 |
| CLPS | PCYT1A |
| SCUBE3 | SNRK |
| POM121L2 | MUC4 |
| MTRF1L | P2RY1 |
| FOXP4 | MYH15 |
| GABRR2 | FAM157A |
| NCR2 | ERICH6 |
| PRICKLE4 | MANF |
| HIST1H1E | PTX3 |
| C6orf165 | MYRIP |
| RAET1L | FRG1 |
| ME1 | WDR19 |
| ATXN1 | CCSER1 |
| FOXC1 | RGS12 |
| C6orf223 | HERC5 |
| FAM46A | ZNF141 |
| MAP3K4 | ANK2 |
| TBP | MYOZ2 |
| HGC6.3 | RNF212 |
| GPR31 | SEC31A |
| RNASET2 | DSPP |
| KMT2C | FAT1 |
| MUC3A | ALPK1 |
| METTL2B | COQ2 |
| FAM20C | TRIML1 |
| PSPH | HMGB2 |
| LRGUK | LRRC66 |
| BBS9 | POU4F2 |
| EGFR | MAML3 |
| GTF2IRD2 | UVSSA |
| FAM185A | MRPS18C |
| STK31 | PF4 |
| MUC17 | ZGRF1 |
| KMT2E | HTT |
| NFE2L3 | CRIPAK |
| PRSS1 | DCHS2 |
| CCZ1 | SDHA |
| MUC12 | ZDHHC11 |
| GNA12 | PCDHA7 |
| CFTR | BRD9 |
| POM121 | MAP3K1 |
| IQCE | RELL2 |
| UPK3BL | PCDHGA2 |
| TTC26 | SLC6A7 |
| STEAP1B | GRXCR2 |
| GSAP | MRPS27 |
| AEBP1 | PCDHGA4 |
| GLI3 | PCDHA6 |
| ZNF736 | ADAM19 |
| COA1 | C5orf42 |
| MICALL2 | NOP16 |
| CPA1 | MYOT |
| PON3 | NSD1 |
| FBXO24 | SEC24A |
| DNAH11 | CDH12 |
| GPR146 | SPEF2 |
| GIMAP8 | CMYA5 |
| RSPH10B, RSPH10B2 | GEMIN5 |
| CTAGE15 | SLC26A2 |
| ELN | BDP1 |
| ABCA13 | KIAA0825 |
| NACAD | TTC37 |
| SSPO | C5orf34 |
| MKRN1 | C5orf45 |
| TRIP6 | PRRC1 |
| CTAGE4 | PCDHA9 |
| FAM115C | ZNF454 |
| OR2A1, OR2A42 | APC |
| TNRC18 | TRIM7 |
| PCLO | TCF7 |
| PODXL | HMGCR |
| GBX1 | ADAMTS6 |
| POMZP3 | MSH3 |
| MEOX2 | C5orf58 |
| DLX6 | MCC |
| LFNG | SLU7 |
| C7orf50 | GPRIN1 |
| RIMS2 | AP3B1 |
| ENPP2 | FAM105A |
| LRRC6 | WWC1 |
| PABPC1 | FAM153B |
| SPAG11B | PRDM9 |
| EYA1 | PCDHB13 |
| PCMTD1 | KCNN2 |
| OXR1 | PCDHB10 |
| CSMD1 | SOWAHA |
| BAI1 | PCDHB2 |
| SORBS3 | PPP2R2B |
| MTUS1 | TMEM232 |
| TIGD5 | RASA1 |
| NEFM | BCLAF1 |
| RP1L1 | TCP10L2 |
| DENND3 | SYNE1 |
| ZNF16 | GPR126 |
| MAPK15 | RHAG |
| TONSL | TCP10 |
| MYOM2 | KCNK16 |
| PARP10 | TAAR6 |
| POLB | HUS1B |
| DEFB104A, DEFB104B | FBXO30 |
| INTS8 | AHI1 |
| FAM83H | CLPS |
| RPL7 | TCF21 |
| FAM150A | TBP |
| DEFB107A, DEFB107B | MAP3K4 |
| CYP11B1 | SHPRH |
| ZNF705G | GPR31 |
| FDFT1 | MDN1 |
| TNFRSF10C | LAMA2 |
| ZFHX4 | FOXP4 |
| MAFA | RNASET2 |
| NAPRT | NCR2 |
| IFNA10 | SCUBE3 |
| PRSS3 | POM121L2 |
| KIAA2026 | MTRF1L |
| OR1L3 | GABRR2 |
| ROR2 | FOXC1 |
| IZUMO3 | ATXN1 |
| FAM205A | PRICKLE4 |
| ANKRD18B | RAET1L |
| CACNA1B | C6orf223 |
| OR13C2 | HIST1H1E |
| CYSRT1 | FAM46A |
| CREB3 | HLA-DRB5 |
| AQP7 | C6orf165 |
| NFX1 | ME1 |
| MAMDC4 | HGC6.3 |
| MPDZ | HLA-DQB1 |
| OR13C5 | LOC441155 |
| NUP188 | PSPH |
| SPATA31C2 | BBS9 |
| WNK2 | KMT2C |
| PPAPDC3 | MUC3A |
| ORM1 | METTL2B |
| NUTM2G | GTF2IRD2 |
| FOXD4 | EGFR |
| NUTM2F | POM121 |
| FIBCD1 | MUC17 |
| ZDHHC12 | PRSS1 |
| PPP1R26 | GNA12 |
| FAM129B | IQCE |
| MED27 | POMZP3 |
| NOXA1 | LRGUK |
| FAM157B | KMT2E |
| IFNA17 | CCZ1 |
| ASPN | UPK3BL |
| LURAP1L | AEBP1 |
| HRCT1 | STK31 |
| SMARCA2 | NFE2L3 |
| UBE2NL | MICALL2 |
| ARSD | MUC12 |
| MAGEB16 | LFNG |
| MAGEE2 | GLI3 |
| EGFL6 | STEAP1B |
| NUP62CL | CPA1 |
| TEX11 | PCLO |
| BRWD3 | FAM185A |
| GPR112 | HOXA5 |
| MAGEA10 | COA1 |
| MAGEC1 | GPR146 |
| AFF2 | GSAP |
| FMR1NB | C7orf50 |
| MXRA5 | ZNF736 |
| ATXN3L | CFTR |
| OR13H1 | FBXO24 |
| MAGEA1 | MEOX2 |
| ZMAT1 | PON3 |
| DMD | TTC26 |
| GAB3 | FAM20C |
| TGIF2LX | GIMAP8 |
| TLR8 | PODXL |
| STARD8 | NACAD |
| VBP1 | ELN |
| WDR44 | GIGYF1 |
| MORC4 | DLX6 |
| P2RY4 | DNAH11 |
| TAB3 | SSPO |
| MAGEB18 | TRIP6 |
| SLC25A43 | CTAGE15 |
| ATRX | RSPH10B, RSPH10B2 |
| GPR174 | MKRN1 |
| GUCY2F | FOXK1 |
| RPGR | ABCA13 |
| SSX5 | FAM115C |
| COL4A6 | PARP12 |
| HDHD1 | OR2A1, OR2A42 |
| MAGIX | LRRC6 |
| MAGEA4 | PABPC1 |
| FAM155B | ENPP2 |
| POF1B | SPAG11B |
| KDM6A | RIMS2 |
| SYTL4 | OXR1 |
| RBMXL3 | TIGD5 |
| GYG2 | RP1L1 |
| RAB40AL | BAI1 |
| CACNA1F | FDFT1 |
| ARMCX5-GPRASP2, GPRASP2 | SORBS3 |
| NHS | EYA1 |
| UTP14A | PARP10 |
| GLRA4 | NEFM |
| BCORL1 | PCMTD1 |
| WDR13 | CSMD1 |
| CXorf36 | TONSL |
| MAGEE1 | MYOM2 |
| FAM47A | DENND3 |
| ATP7A | MAPK15 |
| MAP3K15 | ZNF16 |
| ARMCX4 | FAM83H |
| PRRG3 | MTUS1 |
| TAF7L | INTS8 |
| SLC38A5 | FAM150A |
| MAGEC3 | MAFA |
| KAL1 | POLB |
| FOXR2 | DEFB104A, DEFB104B |
| TBC1D25 | NAPRT |
| POU3F4 | CYP11B1 |
| EDA2R | RPL7 |
| GPR101 | ZFHX4 |
| PAGE2 | PRSS3 |
| ATP11C | ROR2 |
| RAI2 | KIAA2026 |
| FLJ44635 | CACNA1B |
| SLC25A5 | CYSRT1 |
| CXorf40A | FAM205A |
| PNMA3 | OR1L3 |
| ZNF185 | OR13C2 |
| OPN1LW | MAMDC4 |
| MAGEB3 | AQP7 |
| SHROOM2 | ASPN |
| RPL10 | IZUMO3 |
| PSMD10 | LURAP1L |
| XG | NFX1 |
| PLXNB3 | NUP188 |
| CPXCR1 | WNK2 |
| TMEM187 | OR13C5 |
| ARMCX6 | ANKRD18B |
| GABRE | SPATA31C2 |
| HEPH | PPAPDC3 |
| ARSE | ZDHHC12 |
| PRKX | IFNA10 |
| KIAA1210 | SMARCA2 |
| GAGE12J | NUTM2F |
| FAM120C | NUTM2G |
| VCX2 | ORM1 |
| SLC7A3 | FIBCD1 |
| FAM127B | PPP1R26 |
| VCX3A | MPDZ |
| ZBTB33 | FAM129B |
| TCEAL6 | NOXA1 |
| GRIA3 | FAM157B |
| TEX13A; TEX13A | HRCT1 |
| NUDT11 | CLTA |
| PCDH11Y | FOXD4L5 |
| CD24 | MED27 |
|  | CNTNAP3B |
|  | FOXE1 |
|  | IFNA17 |
|  | MAGEA1 |
|  | MAGEC1 |
|  | MXRA5 |
|  | ARSD |
|  | TGIF2LX |
|  | GPR112 |
|  | P2RY4 |
|  | FAM155B |
|  | STARD8 |
|  | MAGEB16 |
|  | MAGEA10 |
|  | AFF2 |
|  | EGFL6 |
|  | NUP62CL |
|  | TEX11 |
|  | SSX5 |
|  | BRWD3 |
|  | MAGIX |
|  | CACNA1F |
|  | OR13H1 |
|  | ZBTB33 |
|  | MAGEB18 |
|  | FAM47A |
|  | WDR13 |
|  | RBMXL3 |
|  | MAGEE1 |
|  | GPR174 |
|  | ZMAT1 |
|  | FMR1NB |
|  | RAB40AL |
|  | POU3F4 |
|  | GUCY2F |
|  | ATXN3L |
|  | DMD |
|  | HDHD1 |
|  | WDR44 |
|  | UBE2NL |
|  | ARMCX4 |
|  | KDM6A |
|  | GLRA4 |
|  | GAB3 |
|  | RPGR |
|  | RAI2 |
|  | TAB3 |
|  | BCORL1 |
|  | MAGEA4 |
|  | SLC38A5 |
|  | GYG2 |
|  | NUDT11 |
|  | TEX13A; TEX13A |
|  | ATRX |
|  | CXorf36 |
|  | SLC25A43 |
|  | TCEAL6 |
|  | MORC4 |
|  | ARMCX5-GPRASP2, GPRASP2 |
|  | TLR8 |
|  | TBC1D25 |
|  | COL4A6 |
|  | PRRG3 |
|  | NHS |
|  | GPR101 |
|  | VBP1 |
|  | POF1B |
|  | UTP14A |
|  | ZNF185 |
|  | MAGEE2 |
|  | TAF7L |
|  | EDA2R |
|  | SYTL4 |
|  | PLXNB3 |
|  | GRIA3 |
|  | KAL1 |
|  | MAP3K15 |
|  | SLC25A5 |
|  | PNMA3 |
|  | FLJ44635 |
|  | FOXR2 |
|  | SHROOM2 |
|  | MAGEB3 |
|  | OPN1LW |
|  | CXorf40A |
|  | ATP11C |
|  | ATP7A |
|  | MAGEC3 |
|  | TMEM187 |
|  | XG |
|  | HEPH |
|  | ARSE |
|  | RPL10 |
|  | PAGE2 |
|  | GABRE |
|  | PRKX |
|  | KIAA1210 |
|  | SLC7A3 |
|  | VCX2 |
|  | CPXCR1 |
|  | ARMCX6 |
|  | FAM120C |
|  | PSMD10 |
|  | GAGE12J |
|  | FAM127B |
|  | VCX3A |
|  | AR |
|  | SLC16A2 |
|  | PCDH11Y |
|  | CD24 |
