# Supplementary material for: Characterization of genetic alterations in brain metastases from non‐small cell lung cancer
Source: FEBS Open Bio. 2018 Aug 30;8(9):1544–52. doi: 10.1002/2211-5463.12501 (PMC6120240; doi:10.1002/2211-5463.12501)
Supplement: Supplementary file 3 — Table S3. Mutant genes identified in P3 primary tumor and matched brain metastasis samples. [file FEB4-8-1544-s003.docx]

**Supplemental table 3. Mutant genes identified in P3 primary tumors and matched brain metastases samples.**

| **P3- primary tumors** | **P3- brain metastases** |
| --- | --- |
| CEP170 | PDE4DIP |
| DNASE2B | MST1L |
| PRAMEF2 | NOTCH2NL |
| PDE4DIP | OR2T34 |
| OR2T5 | CDK11A, CDK11B |
| OR2T35 | HRNR |
| IVL | HNRNPCL1, HNRNPCL3, HNRNPCL4 |
| NOTCH2NL | OR2T35 |
| CDK11B | PER3 |
| HRNR | OR2T3 |
| HNRNPCL1, HNRNPCL3, HNRNPCL4 | OR2T2 |
| OR2T3 | FLG |
| OR2T34 | OR2M5 |
| MST1L | CEP170 |
| XCL1 | FRRS1 |
| OR2T2 | OR2T27 |
| OR2M5 | XCL1 |
| FLG | CDK11B |
| FRRS1 | FCGR3A |
| FCGR3A | ZBED6 |
| ZBED6 | OR2L5 |
| OR2T27 | HNRNPCL2 |
| OR2L5 | PRDM2 |
| HNRNPCL2 | PRAMEF2 |
| CDK11A, CDK11B | F5 |
| F5 | PPIAL4G |
| PRDM2 | TTF2 |
| SHC1 | LCE4A |
| EPHA2 | SHC1 |
| CENPF | OR2T5 |
| PPIAL4G | SPAG17 |
| SSBP3 | EPHA2 |
| AIM1L | NADK |
| SPAG17 | SSBP3 |
| PRAMEF1 | DNASE2B |
| ZMYM6 | NBPF8 |
| TTF2 | PRAMEF1 |
| OBSCN | OXCT2 |
| NBPF8 | ZMYM6 |
| OXCT2 | OR2T4 |
| BMP8B | DDX20 |
| DDX20 | HSPA6 |
| KAZN | RSC1A1 |
| CACNA1S | OBSCN |
| RSC1A1 | AIM1L |
| LAD1 | LAD1 |
| HIVEP3 | CACNA1S |
| HSPA6 | CENPF |
| OR2T4 | HIVEP3 |
| NOL9 | KAZN |
| ATP6V1G3 | ATP6V1G3 |
| CR1L | CR1L |
| ITPKB | DENND4B |
| ZNF692 | GLMN |
| DISP1 | BMP8B |
| DFFA | DISP1 |
| ITLN2 | IGFN1 |
| MEGF6 | ITLN2 |
| PYGO2 | NOL9 |
| LGR6 | ZNF692 |
| CFAP57 | CFAP57 |
| GLMN | DFFA |
| BMP8A | TNN |
| TNN | LGR6 |
| SH3D21 | PYGO2 |
| SELP | AXDND1 |
| DRAXIN | OR4F5 |
| OR2T12 | NAV1 |
| NAV1 | SH3D21 |
| CELA3A | OR2T8 |
| IGFN1 | GDAP2 |
| OR2T8 | SELP |
| OR4F5 | MEGF6 |
| GDAP2 | CDCP2 |
| NASP | CELA3A |
| NBPF10 | OR2T12 |
| MACF1 | BMP8A |
| OR2T33 | ITPKB |
| C1orf64 | DRAXIN |
| LAX1 | PADI4 |
| PADI4 | NASP |
| OR2T29 | SPRR3 |
| KIAA1324 | LAX1 |
| S100PBP | MACF1 |
| SLC35E2 | SLFNL1 |
| SYCP1 | OR2T33 |
| PRAMEF4 | SLC35E2 |
| DNAH14 | C1orf64 |
| PRPF38A | NBPF10 |
| LOC391003, PRAMEF22 | PRPF38A |
| ATAD3B | SYCP1 |
| NOTCH2 | OR2T29 |
| LOC101929983, PRAMEF6, PRAMEF9 | KIAA1324 |
| HECTD3 | CRP |
| PLEKHG5 | ATAD3B |
| CROCC | S100PBP |
| PRDM16 | MIR205HG |
| NBPF15 | LOC101929983, PRAMEF6, PRAMEF9 |
| SLC35E2B | LOC391003, PRAMEF22 |
| CELSR2 | IVL |
| PYCR2 | INPP5B |
| NBPF14, NBPF8, NBPF9 | KCNN3 |
| INPP5B | PRDM16 |
| RBMXL1 | CROCC |
| TESK2 | NOTCH2 |
| FAM131C | DNAH14 |
| LCE4A | NBPF14, NBPF8, NBPF9 |
| NADK | PRAMEF4 |
| KCNN3 | SLC35E2B |
| PER3 | NBPF15 |
| DENND4B | HECTD3 |
| AXDND1 | PLEKHG5 |
| SLFNL1 | CELSR2 |
| SPRR3 | PYCR2 |
| MIR205HG | FMN2 |
| FMN2 | DMRTA2 |
| CDCP2 | TESK2 |
| LOC100996758, NPY4R | FRG2B |
| FRG2B | SYT15 |
| SYT15 | LOC100996758, NPY4R |
| GPRIN2 | EIF5AL1 |
| CDHR1 | GPRIN2 |
| TIMM23 | FBXW4 |
| EIF5AL1 | LYZL2 |
| FBXW4 | CDHR1 |
| RTKN2 | RTKN2 |
| SVIL | TIMM23 |
| ZNF239 | ZNF239 |
| LYZL2 | AFAP1L2 |
| WDFY4 | SVIL |
| C10orf12 | WDFY4 |
| AGAP9 | ARID5B |
| PLCE1 | TRIM8 |
| DMBT1 | PLCE1 |
| TRIM8 | C10orf12 |
| PDZD7 | AGAP9 |
| LARP4B | DMBT1 |
| NCOA4 | NCOA4 |
| BMS1 | PDZD7 |
| BICC1 | LARP4B |
| SLK | BMS1 |
| NRAP | SLK |
| DRGX | BICC1 |
| GDF2 | DCLRE1C |
| TUBB8 | NRAP |
| DCLRE1C | GDF2 |
| PDLIM1 | DRGX |
| RRP12 | RRP12 |
| CUBN | AGAP7P |
| AGAP7P | PDLIM1 |
| HELLS | CUBN |
| COMTD1 | COMTD1 |
| AFAP1L2 | SAA2, SAA2-SAA4 |
| ARID5B | BTG4 |
| OR4C3 | OR8U1, OR8U8 |
| SAA2, SAA2-SAA4 | OR8B2 |
| OR9G1, OR9G9 | OR9G1, OR9G9 |
| OR8U1, OR8U8 | OR4C3 |
| OR8B2 | OR8B3 |
| BTG4 | OR8G2 |
| OR8B3 | CNTN5 |
| OR8G2 | MUC5B |
| MUC6 | OR51F1 |
| TRMT112 | OR8D4 |
| OR51F1 | CDON |
| MUC5B | FIBIN |
| CNTN5 | OR8K1 |
| OR8K1 | ARHGAP32 |
| FIBIN | TRMT112 |
| OR8D4 | NPAT |
| ARHGAP32 | OR8D2 |
| OR10G4 | OR10G4 |
| OR8D2 | MUC6 |
| CDON | OR8U1, OR8U8 |
| ST5 | ZNF202 |
| NPAT | OR4D6 |
| ALDH3B2 | OR51Q1 |
| OR8U1, OR8U8 | ST5 |
| MAML2 | OR51G2 |
| ZNF202 | C11orf40 |
| OR51Q1 | GLYATL1 |
| NUP98 | ALDH3B2 |
| OR4D6 | NUP98 |
| OR4C16 | OR4C16 |
| OR8D1 | OR8D1 |
| OR4D5 | TRIM49 |
| OR51G2 | OR4D5 |
| OTOG | MAML2 |
| FGF3 | FGF3 |
| TRIM49 | TMPRSS13 |
| ZNHIT2 | OTOG |
| TH | MRGPRX3 |
| GLYATL1 | ZNHIT2 |
| ALX4 | TH |
| MRGPRX3 | TENM4 |
| OR51A4 | OR51A4 |
| MOGAT2 | MOGAT2 |
| PHLDB1 | PHLDB1 |
| OR1S1 | CD248 |
| C2CD2L | CNTF |
| TENM4 | OR8U1 |
| OR8U1 | OR1S1 |
| CNTF | ALX4 |
| AP5B1 | DCHS1 |
| ESRRA | KRTAP5-5 |
| MUC2 | C2CD2L |
| OR4B1 | AP5B1 |
| ANO5 | RAPSN |
| EXPH5 | OR4B1 |
| SAA1 | OR8G5 |
| SPI1 | EXPH5 |
| FUT4 | KRTAP5-7 |
| NAALAD2 | KRTAP5-2 |
| KRTAP5-1 | ANO5 |
| SNX19 | MICALCL |
| TRIM49C | C11orf80 |
| BRSK2 | ESRRA |
| KRTAP5-5 | SAA1 |
| DGKZ | SPI1 |
| DCHS1 | TRIM49C |
| C11orf80 | NAALAD2 |
| KRTAP5-7 | SNX19 |
| MICALCL | KRTAP5-1 |
| TMPRSS13 | TAS2R46 |
| CD248 | TAS2R43 |
| KRTAP5-2 | TAS2R31 |
| C11orf40 | TAS2R19 |
| TAS2R19 | TAS2R30 |
| TAS2R43 | C12orf56 |
| TAS2R31 | KRT6A |
| TAS2R46 | RASSF8 |
| C12orf56 | SLC38A4 |
| GXYLT1 | PRH2 |
| TAS2R30 | KRT6B |
| KRT6A | KERA |
| SLC38A4 | OR6C3 |
| PRH2 | DCP1B |
| RASSF8 | KMT2D |
| KERA | PRPF40B |
| KRT6B | CMAS |
| PRPF40B | ATN1 |
| OR6C3 | PTPRB |
| RBM19 | CS |
| PTPRB | USP44 |
| KMT2D | PAH |
| USP44 | KRT3 |
| CMAS | RBM19 |
| PAH | SDR9C7 |
| SDR9C7 | EP400 |
| EP400 | PHLDA1 |
| SLC2A3 | SLC2A3 |
| NANOGNB | KLRC2 |
| KLRC2 | C12orf42 |
| VWF | NANOGNB |
| C12orf42 | VWF |
| KRT73 | KRT73 |
| FAM186A | FAM109A |
| FAM109A | CELA1 |
| PARPBP | PARPBP |
| FBRSL1 | WDR66 |
| KRT18 | PRB1 |
| PIP4K2C | KRT2 |
| CCDC63 | PIP4K2C |
| KLRC3 | FBRSL1 |
| HCAR3 | FAM186A |
| DCP1B | KRT18 |
| KRT2 | KLRC3 |
| WDR66 | ASCL1 |
| BRI3BP | HCAR3 |
| GDF11 | CCDC63 |
| KRT3 | BRI3BP |
| ATN1 | TPTE2 |
| PHLDA1 | SKA3 |
| ZNF384 | MTMR6 |
| PEX5 | C1QTNF9 |
| CELA1 | CCDC168 |
| TDG | PSPC1 |
| CAMKK2 | MRPL57 |
| PRB3 | FREM2 |
| SKA3 | ATP7B |
| PARP4 | DGKH |
| MRPL57 | CDX2 |
| TPTE2 | VWA8 |
| MTMR6 | PARP4 |
| C1QTNF9 | FLT3 |
| PSPC1 | IFT88 |
| CCDC168 | DACH1 |
| ATP7B | NUDT15 |
| FREM2 | WDR89 |
| CDX2 | OR11G2 |
| DGKH | RBM23 |
| VWA8 | AHNAK2 |
| FLT3 | HSP90AA1 |
| IFT88 | GOLGA5 |
| NUDT15 | AKAP6 |
| DACH1 | PNN |
| SLAIN1 | MDGA2 |
| WDR89 | CLMN |
| AHNAK2 | PCK2 |
| GOLGA5 | NYNRIN |
| AKAP6 | HOMEZ |
| PNN | REC8 |
| NYNRIN | SIPA1L1 |
| PCK2 | IPO4 |
| SIPA1L1 | TMEM30B |
| MDGA2 | ALDH6A1 |
| CLMN | MMP14 |
| HOMEZ | OR4N2 |
| TMEM30B | ATXN3 |
| IPO4 | NOP9 |
| MMP14 | RALGAPA1 |
| ALDH6A1 | CEP170B |
| OR4N2 | CHGA |
| RALGAPA1 | ADAM21 |
| POTEG | POTEG |
| CEP170B | IRF2BPL |
| CHGA | C14orf105 |
| ADAM21 | MAP3K9 |
| C14orf105 | GOLGA8R |
| ACOT2 | OR4M2 |
| OR10G2 | OR4N4 |
| RBM23 | ZSCAN2 |
| REC8 | STARD9 |
| NOP9 | C15orf39 |
| HSP90AA1 | HERC2 |
| MAP3K9 | TRPM1 |
| IRF2BPL | IQCH |
| OR11G2 | ZNF609 |
| ATXN3 | MCTP2 |
| OR4M2 | VPS13C |
| OR4N4 | MEF2A |
| IQCH | CIB1 |
| ZSCAN2 | DYX1C1 |
| STARD9 | C15orf40 |
| HERC2 | SEMA7A |
| ZNF609 | MAPKBP1 |
| CIB1 | OCA2 |
| MAPKBP1 | MESP2 |
| MCTP2 | CATSPER2 |
| VPS13C | FAN1 |
| DYX1C1 | POTEB, POTEB2, POTEB3 |
| OCA2 | TJP1 |
| MESP2 | TGM5 |
| SEMA7A | SORD |
| CATSPER2 | ADAMTS7 |
| FAN1 | GOLGA6A |
| TJP1 | CHRNA3 |
| POTEB, POTEB2, POTEB3 | GOLGA8H |
| TGM5 | GOLGA8A |
| SORD | PHKB |
| ADAMTS7 | CES1 |
| GOLGA6A | UBN1 |
| C2CD4A | RRN3 |
| GOLGA8A | MPHOSPH6 |
| GOLGA6L1, GOLGA6L22 | NLRC5 |
| C15orf39 | E2F4 |
| MEF2A | IL32 |
| CHRNA3 | OTOA |
| TRPM1 | TNRC6A |
| OTOA | PALB2 |
| PHKB | ESRP2 |
| RRN3 | FTO |
| CES1 | SULT1A1 |
| UBN1 | CYB5B |
| NLRC5 | PKD1L2 |
| SULT1A1 | ZNF646 |
| TNRC6A | SLC7A6 |
| MPHOSPH6 | PRR25 |
| IL32 | MLYCD |
| ESRP2 | PDXDC1 |
| PALB2 | PHLPP2 |
| PDPR | SMPD3 |
| CYB5B | TPSAB1 |
| PRR25 | CLEC18B |
| ZNF646 | ACSM5 |
| FTO | SLC12A4 |
| SMPD3 | GLYR1 |
| MLYCD | ZFP90 |
| SLC7A6 | ZFHX3 |
| PDXDC1 | JPH3 |
| PHLPP2 | TPSD1 |
| GLYR1 | CNOT1 |
| CACNA1H | CACNA1H |
| TPSAB1 | MSLN |
| CLEC18B | ADAMTS18 |
| ACSM5 | CLEC18C |
| MSLN | ZNF469 |
| TPSD1 | TPSB2 |
| ZNF469 | SNAI3 |
| CLEC18C | RPUSD1 |
| ZFP90 | CNGB1 |
| TPSB2 | APOBR |
| ADAMTS18 | MAP2K3 |
| SNAI3 | KCNJ12, KCNJ18 |
| CNGB1 | TMEM104 |
| LOC100129697 | FBXW10 |
| CYBA | MLLT6 |
| ZFHX3 | OR1D5 |
| XYLT1 | SARM1 |
| E2F4 | TVP23C, TVP23C-CDRT4 |
| SLC12A4 | CCDC144NL |
| JPH3 | MYO15A |
| APOBR | USP6 |
| PKD1L2 | KRTAP1-1 |
| CNOT1 | C17orf104 |
| MAP2K3 | KRTAP4-9 |
| DNAH2 | RNF43 |
| NCOR1 | MYH2 |
| KCNJ12, KCNJ18 | TRIM47 |
| TMEM104 | PHF12 |
| MLLT6 | BPTF |
| FBXW10 | RAI1 |
| MYO15A | TEKT1 |
| OR1D5 | KRTAP4-7 |
| KRTAP1-1 | QRICH2 |
| CCDC144NL | UTS2R |
| USP6 | ABCA9 |
| RNF43 | MPRIP |
| TVP23C, TVP23C-CDRT4 | FADS6 |
| KRTAP4-9 | DNAH2 |
| C17orf104 | NCOR1 |
| TRIM47 | NACA2 |
| PHF12 | EVPL |
| MYH2 | KRTAP4-1 |
| UTS2R | CNTD1 |
| KRTAP4-7 | KRTAP4-6 |
| TEKT1 | LRRC37B |
| CNTD1 | LRRC37A |
| ABCA9 | KRTAP9-2 |
| NACA2 | SLFN13 |
| KRTAP4-12 | KRTAP4-12 |
| KRTAP4-6 | KDM6B |
| LRRC37A | TBC1D28 |
| KRTAP9-2 | HELZ |
| LRRC37B | KRTAP1-3 |
| SLFN13 | INTS2 |
| CSH1 | KRTAP4-8 |
| TBC1D28 | GSDMA |
| HELZ | CCDC57 |
| CDC27 | CDC27 |
| GSDMA | C17orf97 |
| ALOX15B | ROCK1 |
| INTS2 | TXNDC2 |
| CCDC57 | C18orf63 |
| KRTAP4-11 | EMILIN2 |
| KRT10 | SALL3 |
| FADS6 | POTEC |
| BPTF | CEP192 |
| KRTAP1-3 | KATNAL2 |
| KDM6B | ANKRD30B |
| MPRIP | EPG5 |
| QRICH2 | CNDP1 |
| KRTAP4-1 | MUC16 |
| C17orf97 | LILRA6, LILRB3 |
| SARM1 | RFPL4A |
| RAI1 | ZNF561 |
| ROCK1 | NLRP7 |
| TXNDC2 | ZNF460 |
| C18orf63 | URI1 |
| SALL3 | ZNF761 |
| EMILIN2 | ZNF320 |
| POTEC | APC2 |
| MYO5B | WDR87 |
| CEP192 | RHPN2 |
| KATNAL2 | SLC1A5 |
| EPG5 | PLIN4 |
| ANKRD30B | ARHGAP35 |
| CNDP1 | FBN3 |
| RHPN2 | ZNF600 |
| ZNF506 | ZNF486 |
| KLK10 | AZU1 |
| LILRA6, LILRB3 | RFPL4AL1 |
| ZNF561 | ZNF527 |
| MUC16 | PSG3 |
| ZNF761 | FBL |
| NLRP7 | RYR1 |
| ZNF320 | PKN1 |
| ZNF460 | CCDC114 |
| FBN3 | LENG8 |
| WDR87 | ICAM3 |
| ZNF486 | FFAR3 |
| PKN1 | ZNF626 |
| RFPL4AL1 | OR10H1 |
| SLC1A5 | ZBTB32 |
| AZU1 | PCP2 |
| ARHGAP35 | ZNF565 |
| ZNF600 | C19orf38 |
| PCP2 | TICAM1 |
| PLIN4 | CPT1C |
| RFPL4A | CIC |
| ICAM3 | ZNF506 |
| CPT1C | ZNF317 |
| TICAM1 | ARHGAP33 |
| ZBTB32 | DNMT1 |
| FFAR3 | ZNF708 |
| RYR1 | ZNF302 |
| ZNF626 | TIMM50 |
| FBL | FCGBP |
| LENG8 | CREB3L3 |
| ARHGAP33 | C19orf35 |
| ZNF317 | PDE4A |
| FCGBP | TYK2 |
| C19orf38 | YJEFN3 |
| PSG3 | HDGFRP2 |
| PDE4A | EPN1 |
| DNMT1 | CLEC4G |
| C19orf35 | DOCK6 |
| ZNF565 | CRLF1 |
| TIMM50 | ZNF83 |
| CIC | SIGLEC11 |
| ZNF708 | KLK10 |
| ZNF302 | ATF5 |
| CREB3L3 | SBK2 |
| EMR2 | ANKLE1 |
| HDGFRP2 | SSC5D |
| YJEFN3 | GRIK5 |
| TYK2 | HSPBP1 |
| GRIK5 | DUXA |
| SBK2 | RFX2 |
| EPN1 | SDHAF1 |
| CLEC4G | MAP3K10 |
| SIGLEC11 | RSPH6A |
| DOCK6 | KIR2DL1 |
| CRLF1 | CNN2 |
| ATF5 | MFSD12 |
| SSC5D | KIR3DS1 |
| SDHAF1 | KIR2DS1 |
| MAP3K10 | IL1RL1 |
| DUXA | MYO3B |
| ANKLE1 | LRP1B |
| KIR2DL1 | EDAR |
| MFSD12 | TTN |
| MADCAM1 | INPP1 |
| CNN2 | ITPRIPL1 |
| KCNC3 | ALMS1 |
| HSPBP1 | SPATA3 |
| RSPH6A | CHAC2 |
| URI1 | ANKRD36 |
| ZNF83 | CPS1 |
| CCDC114 | USP40 |
| ZNF527 | ATP5G3 |
| KIR3DS1 | ARMC9 |
| KIR2DS1 | LRP2 |
| IL1RL1 | TUBA3E |
| MYO3B | ASXL2 |
| TTN | FRZB |
| ALMS1 | EMILIN1 |
| ANKRD36 | LRRTM1 |
| INPP1 | HOXD9 |
| LRP1B | INO80D |
| EDAR | AFF3 |
| ITPRIPL1 | REG3A |
| CPS1 | CYP26B1 |
| CHAC2 | NOSTRIN |
| USP40 | PRR30 |
| TUBA3E | FAM171B |
| ARMC9 | CCDC74A |
| EMILIN1 | C2orf70 |
| ASXL2 | SP110 |
| CYP26B1 | OR6B3 |
| LRRTM1 | IFT172 |
| LRP2 | TEKT4 |
| INO80D | GCKR |
| FRZB | GPD2 |
| ATP5G3 | OSBPL6 |
| AFF3 | APOB |
| REG3A | COL6A3 |
| PRR30 | SLC16A14 |
| SP110 | CCDC74B |
| NOSTRIN | C2orf73 |
| OR6B3 | HSPD1 |
| IFT172 | SCN7A |
| CCDC74A | HS6ST1 |
| GCKR | CCNYL1 |
| SLC16A14 | EML4 |
| CCDC74B | POTEJ |
| APOB | SMYD1 |
| HSPD1 | POTEE |
| OSBPL6 | POTEF |
| GPD2 | TUBA3D |
| COL6A3 | TMEM127 |
| SCN7A | SP5 |
| POTEJ | TTC30A |
| CCNYL1 | DEFB126 |
| TMEM127 | TMEM74B |
| C2orf73 | SEMG1 |
| EML4 | MYT1 |
| POTEE | TUBB1 |
| POTEF | DEFB132 |
| SMYD1 | SLC17A9 |
| TUBA3D | APCDD1L |
| HS6ST1 | NAPB |
| TTC30A | CTSA |
| FAM171B | SIRPA |
| SPATA3 | PRNP |
| HOXD9 | RALY |
| C2orf70 | HELZ2 |
| TMEM74B | NPEPL1 |
| SEMG1 | BAGE2, BAGE3 |
| MYT1 | TPTE |
| TUBB1 | URB1 |
| SLC17A9 | IL10RB |
| APCDD1L | KRTAP10-1 |
| NAPB | DOPEY2 |
| HELZ2 | PRDM15 |
| PRNP | KRTAP10-7 |
| SIRPA | TMPRSS3 |
| NPEPL1 | TRPM2 |
| RALY | COL18A1 |
| DEFB132 | DEPDC5 |
| CTSA | SLC5A4 |
| DEFB126 | NEFH |
| TPTE | ZNF70 |
| BAGE2, BAGE3 | RFPL3 |
| URB1 | EIF4ENIF1 |
| IL10RB | TRIOBP |
| DOPEY2 | CYP2D6 |
| PRDM15 | PRAME |
| KRTAP10-7 | SFI1 |
| TMPRSS3 | SUSD2 |
| TRPM2 | DGCR8 |
| KRTAP10-6 | GSTT2, GSTT2B |
| COL18A1 | GGT1 |
| KRTAP10-1 | POLR2F |
| SLC5A4 | PLA2G6 |
| DEPDC5 | SRRD |
| ZNF70 | MLC1 |
| RFPL3 | NEK4 |
| TRIOBP | MUC20 |
| EIF4ENIF1 | C3orf17 |
| PRAME | LRIG1 |
| SFI1 | LMLN |
| SUSD2 | KNG1 |
| GSTT2, GSTT2B | P2RY14 |
| GGT1 | LAMP3 |
| DGCR8 | ACOX2 |
| POLR2F | GOLGA4 |
| PLA2G6 | MCM2 |
| NEFH | CLSTN2 |
| SRRD | CD80 |
| BCR | PIK3R4 |
| ILDR1 | TRAK1 |
| ZNF717 | PLXNB1 |
| MUC20 | FBXO40 |
| C3orf17 | TOPAZ1 |
| NEK4 | ILDR1 |
| LMLN | ZNF717 |
| LRIG1 | ATG3 |
| MCM2 | ZBBX |
| KNG1 | KLHL24 |
| P2RY14 | TOMM70A |
| LAMP3 | LNP1 |
| CLSTN2 | CASR |
| PIK3R4 | MED12L |
| GOLGA4 | SLC38A3 |
| CD80 | EFCAB12 |
| PLXNB1 | KIAA2018 |
| TOPAZ1 | KIAA1143 |
| PTPN23 | CSRNP1 |
| KIAA1143 | PTPN23 |
| SLC38A3 | CDHR4 |
| ZBBX | ANKUB1 |
| CSRNP1 | RPL14 |
| CASR | MAGEF1 |
| KLHL24 | CTNNB1 |
| CDHR4 | CCDC66 |
| MED12L | ALG3 |
| EFCAB12 | MAGI1 |
| MST1 | SFMBT1 |
| ALG3 | RBM5 |
| SFMBT1 | MST1 |
| MUC4 | TMIE |
| COPB2 | EPHA3 |
| DZIP1L | ERICH6 |
| MAATS1 | DZIP1L |
| EPHA3 | COPB2 |
| TPRA1 | MAATS1 |
| MANF | MUC4 |
| TRAK1 | TPRA1 |
| LNP1 | FAM157A |
| MAGEF1 | MANF |
| RPL14 | FRG1 |
| CCDC66 | BST1 |
| MAGI1 | KLF3 |
| KIAA2018 | KLB |
| TMIE | SPARCL1 |
| FAM157A | CRMP1 |
| ATG3 | CCKAR |
| FBXO40 | SORBS2 |
| ANKUB1 | ZNF141 |
| RBM5 | HTT |
| THAP9 | WWC2 |
| FRG1 | MAML3 |
| BST1 | ZNF732 |
| KLF3 | TBC1D9 |
| KLB | MRPL1 |
| WWC2 | DSPP |
| CRMP1 | POU4F2 |
| CCKAR | RNF212 |
| SPARCL1 | THAP9 |
| SORBS2 | SRP72 |
| ZNF141 | HPGD |
| ZNF732 | ACSL1 |
| HTT | APBB2 |
| MAML3 | DCHS2 |
| TBC1D9 | PHOX2B |
| MRPL1 | SH3TC1 |
| SRP72 | FGFR3 |
| HPGD | NKX3-2 |
| DSPP | MRPS27 |
| APBB2 | SDHA |
| ACSL1 | ZDHHC11 |
| FGFR3 | GABRP |
| SH3TC1 | MAP3K1 |
| NKX3-2 | GPR98 |
| POU4F2 | CARD6 |
| RNF212 | C6 |
| PHOX2B | PCDHGA12 |
| DCHS2 | C5orf49 |
| C5orf49 | MYOT |
| MRPS27 | EPB41L4A |
| GABRP | NOP16 |
| CARD6 | SLC22A4 |
| GPR98 | NDUFA2 |
| PCDHGA12 | PRDM6 |
| C6 | MXD3 |
| NDUFA2 | CMYA5 |
| MYOT | AQPEP |
| EPB41L4A | ERBB2IP |
| ERBB2IP | PCDHA7 |
| PRDM6 | SLC26A2 |
| AQPEP | HSD17B4 |
| PCDHA7 | PCDH12 |
| BRD9 | SLC6A7 |
| C5orf45 | ADAMTS16 |
| CMYA5 | AP3B1 |
| SLC26A2 | KIF20A |
| SLC9A3 | C5orf45 |
| SLC6A7 | SLC9A3 |
| PCDH12 | MSH3 |
| KIF20A | IL7R |
| HSD17B4 | DIAPH1 |
| DIAPH1 | TTC33 |
| AP3B1 | KCNN2 |
| IL7R | SPEF2 |
| TTC33 | PAM |
| SPEF2 | PCDHA9 |
| PCDHA9 | SLU7 |
| SOWAHA | C5orf58 |
| PAM | SOWAHA |
| PRDM9 | FAM153B |
| C5orf58 | PCDHA8 |
| SLU7 | SLC12A2 |
| PCDHA8 | PRDM9 |
| FAM153B | WWC1 |
| PCDHB12 | PCDHB12 |
| MSH3 | TCP10 |
| KCNN2 | BCLAF1 |
| SLC12A2 | GPR116 |
| MAST4 | MAK |
| MAP3K1 | FAM120B |
| SLC22A4 | SYNE1 |
| WWC1 | RREB1 |
| NOP16 | USP49 |
| SDHA | BTN3A2 |
| MXD3 | CUL7 |
| TCP10L2 | HMGCLL1 |
| BTN3A3 | TCP10L2 |
| BCLAF1 | BTN3A3 |
| TCP10 | KIAA1244 |
| MAK | ECI2 |
| SYNE1 | LOC441155 |
| RREB1 | MAP3K4 |
| USP49 | BMP6 |
| CUL7 | ZNF322 |
| KIAA1244 | ITPR3 |
| BTN3A2 | TFEB |
| ECI2 | PKHD1 |
| HMGCLL1 | ATXN1 |
| LOC441155 | MTRF1L |
| PKHD1 | TBP |
| ZNF322 | BCLAF1; BCLAF1 |
| ITPR3 | RIOK1 |
| TFEB | FIG4 |
| MTRF1L | UFL1 |
| FIG4 | MTFR2 |
| ATXN1 | LMBRD1 |
| MTFR2 | PLG |
| RIOK1 | HLA-DRB5 |
| HLA-DRB5 | C6orf223 |
| UFL1 | HLA-DQB1 |
| LMBRD1 | CYP21A2 |
| PLG | HLA-A |
| HLA-DQB1 | PSPH |
| CYP21A2 | SMKR1 |
| HLA-A | KMT2C |
| FAM120B | MUC3A |
| BMP6 | GTF2IRD2, GTF2IRD2B |
| BCLAF1; BCLAF1 | METTL2B |
| C6orf223 | BBS9 |
| FAM46A | MUC17 |
| SLC35D3 | SAMD9L |
| GPR116 | PRSS1 |
| MAP3K4 | ZFAND2A |
| TBP | TARP |
| TCP11 | HEATR2 |
| KMT2C | POM121 |
| MUC3A | GALNT11 |
| FAM20C | IQCE |
| PSPH | KIAA1549 |
| SMKR1 | COBL |
| GTF2IRD2, GTF2IRD2B | CHST12 |
| METTL2B | LIMK1 |
| BBS9 | DOCK4 |
| MUC17 | RASA4, RASA4B |
| SAMD9L | STEAP1B |
| IQCE | CROT |
| POM121 | CARD11 |
| ZFAND2A | SCRN1 |
| PRSS1 | STK31 |
| HEATR2 | MUC12 |
| CHST12 | LFNG |
| TARP | SUMF2 |
| GALNT11 | FAM185A |
| KIAA1549 | CFTR |
| STK31 | FASTK |
| DOCK4 | FTSJ2 |
| MUC12 | INTS1 |
| FASTK | PCLO |
| FAM185A | DAGLB |
| RASA4, RASA4B | FAM20C |
| CARD11 | TBRG4 |
| SUMF2 | C7orf50 |
| SCRN1 | MEOX2 |
| CROT | ZNF138 |
| LIMK1 | RADIL |
| STEAP1B | SSBP1 |
| FTSJ2 | GTF2IRD2 |
| CFTR | PODXL |
| INTS1 | SUN1 |
| C7orf50 | GRB10 |
| DAGLB | CTAGE15 |
| TBRG4 | SHH |
| RADIL | GPR141 |
| GTF2IRD2 | ZPBP |
| ZNF138 | TMEM184A |
| SSBP1 | FAM115C |
| SUN1 | AKAP9 |
| GRB10 | NSMAF |
| ZPBP | SPAG11B |
| GPR141 | RB1CC1 |
| SHH | RP1L1 |
| FAM115C | EPPK1 |
| CTAGE4, CTAGE8 | ZNF696 |
| CTAGE15 | CDH17 |
| PCLO | POP1 |
| PODXL | VPS13B |
| TMEM184A | RECQL4 |
| COBL | NPBWR1 |
| MEOX2 | FDFT1 |
| LFNG | PKHD1L1 |
| NSMAF | CSMD1 |
| SPAG11B | PCMTD1 |
| RB1CC1 | KIF13B |
| ZNF696 | GINS4 |
| RECQL4 | PEBP4 |
| EPPK1 | ANXA13 |
| NPBWR1 | PIWIL2 |
| VPS13B | C8orf58 |
| PKHD1L1 | MAFA |
| POP1 | DEFB107A, DEFB107B |
| KIF13B | DEFB104A, DEFB104B |
| RP1L1 | PRSS3 |
| PCMTD1 | OR13C2 |
| PEBP4 | CACNA1B |
| CSMD1 | OR13C5 |
| GINS4 | CNTRL |
| ANXA13 | APBA1 |
| PIWIL2 | CIZ1 |
| C8orf58 | MAMDC4 |
| DEFB107A, DEFB107B | ALDH1A1 |
| DEFB104A, DEFB104B | ASPN |
| FDFT1 | TRAF1 |
| MAFA | INVS |
| IFNA10 | SURF6 |
| PRSS3 | C9orf50 |
| OR13C2 | AQP7 |
| CACNA1B | LURAP1L |
| APBA1 | NUTM2F |
| MAMDC4 | C9orf135 |
| CNTRL | KIF24 |
| OR13C5 | IFNA10 |
| INVS | SDCCAG3 |
| AQP7 | RASEF |
| ALDH1A1 | ORM1 |
| CIZ1 | NUTM2G |
| TRAF1 | SNAPC4 |
| SURF6 | ENTPD2 |
| C9orf50 | NOL8 |
| C9orf135 | GLIS3 |
| NUTM2F | SEC16A |
| SDCCAG3 | FAM157B |
| KIF24 | FOXE1 |
| NUTM2G | FOXD4 |
| RASEF | FOXD4L5 |
| ORM1 | CNTNAP3B |
| SNAPC4 | ANKRD20A2, ANKRD20A3 |
| ENTPD2 | IFNA17 |
| GLIS3 | MAGEA10 |
| FOXD4 | MAGEC1 |
| NOL8 | NUP62CL |
| SEC16A | GPR112 |
| FOXD4L5 | OR13H1 |
| ANKRD20A2, ANKRD20A3 | ZNF645 |
| CNTNAP3B | ZBTB33 |
| WDR34 | RBMXL3 |
| IFNA17 | ZMAT1 |
| LURAP1L | FGF13 |
| FAM157B | TAZ |
| ASPN | GUCY2F |
| FOXE1 | TGIF2LX |
| UBE2NL | SUPT20HL1 |
| ARSD | TEX13A, TEX13A |
| MAGEE2 | TCEAL6 |
| MAGEB16 | GPR174 |
| RLIM | IRS4 |
| MAGEA10 | ARSD |
| MAGEC1 | RBMX |
| NUP62CL | MAGEB16 |
| GPR112 | UBE2NL |
| OR13H1 | MAGEA1 |
| TGIF2LX | BCORL1 |
| FAM155B | TCEANC |
| ZNF645 | MORC4 |
| RBMXL3 | FMR1NB |
| TAZ | GLRA4 |
| SUPT20HL1 | WDR13 |
| FGF13 | MAGEB18 |
| GLRA4 | BRWD3 |
| ZMAT1 | MAGIX |
| FMR1NB | GPR101 |
| BCORL1 | ZCCHC16 |
| SSX5 | VBP1 |
| MAGEA1 | DMD |
| VBP1 | FAM155B |
| MAGIX | MAGEA4 |
| RBMX | PRRG3 |
| GUCY2F | COL4A6 |
| IRS4 | SSX5 |
| GPR174 | ZFX |
| SLC25A43 | ARMCX4 |
| BRWD3 | SLC25A43 |
| TCEANC | SYTL4 |
| COL4A6 | TNMD |
| MORC4 | ATXN3L |
| MAGEB18 | GPC4 |
| WDR13 | POU3F4 |
| ARMCX4 | TLR8 |
| PRRG3 | GYG2 |
| DMD | MAP7D2 |
| ZCCHC16 | TAF7L |
| TNMD | TBC1D25 |
| ATXN3L | MED12 |
| GPR101 | TAB3 |
| GYG2 | DDX53 |
| MAMLD1 | CXorf40A |
| MAGEA4 | MAMLD1 |
| SATL1 | POF1B |
| GPC4 | SATL1 |
| DDX53 | RPS6KA6 |
| MAP7D2 | NUDT11 |
| POU3F4 | PLXNB3 |
| MED12 | CYLC1 |
| CXorf40A | PNMA3 |
| PLXNB3 | SLC25A5 |
| TLR8 | MAGEC3 |
| MAGEC3 | RAI2 |
| TAB3 | MAP3K15 |
| TAF7L | PRR32 |
| RAI2 | WWC3 |
| POF1B | MAGEE2 |
| SOWAHD | RPA4 |
| RPS6KA6 | SOWAHD |
| RPA4 | KAL1 |
| PRR32 | GRIA3 |
| TBC1D25 | TMEM187 |
| SYTL4 | RGAG4 |
| TMEM187 | RPL10 |
| CYLC1 | BEND2 |
| WWC3 | GLA |
| PNMA3 | FOXR2 |
| EDA2R | DCAF8L2 |
| KAL1 | EDA2R |
| FOXR2 | HEPH |
| RGAG4 | GABRE |
| MAP3K15 | MAGEB3 |
| PRKX | MAGEB2 |
| RPL10 | PRKX |
| BEND2 | ATP7A |
| GLA | FLJ44635 |
| MAGEB3 | XG |
| PAGE2 | ATP11C |
| GABRE | PAGE2 |
| FAM120C | ARSE |
| ATP7A | FAM120C |
| SLC25A5 | CXorf58 |
| ATP11C | KIAA1210 |
| MAGEB2 | CPXCR1 |
| FLJ44635 | VCX3A |
| XG | SLC7A3 |
| ARSE | NLGN4X |
| CPXCR1 | VCX2 |
| HCFC1 | MID1 |
| HEPH | HCFC1 |
| VCX2 | SSX4, SSX4B |
| NLGN4X | PCDH11Y |
| SLC7A3 | CD24 |
| PIN4 |  |
| KIAA1210 | |
| VCX3A |  |
| MID1 |  |
| ZBTB33 |  |
| DCAF8L2 | |
| TCEAL6 |  |
| GRIA3 |  |
| TEX13A, TEX13A | |
| NUDT11 |  |
| PCDH11Y | |
| CD24 |  |
