# Supplementary material for: Characterization of genetic alterations in brain metastases from non‐small cell lung cancer
Source: FEBS Open Bio. 2018 Aug 30;8(9):1544–52. doi: 10.1002/2211-5463.12501 (PMC6120240; doi:10.1002/2211-5463.12501)
Supplement: Supplementary file 4 — Table S4. Mutant genes identified in P4 primary tumor and matched brain metastasis samples. [file FEB4-8-1544-s004.docx]

**Supplemental table 4. Mutant genes identified in P4 primary tumors and matched brain metastases samples.**

| **P4- primary tumors** | **P4- brain metastases** |
| --- | --- |
| EGFR | TCF7L2 |
| KMT2C | RUNX1 |
| PRKDC | ADAM29 |
| RUNX1 | RPTOR |
| FANCD2 | LRP1B |
| ATRX | KMT2C |
| FAM135B | TSC1 |
| RGPD4 | PRKDC |
| RGPD3 | TSC1 |
| BIRC5 | KMT2C |
| NF1 | PKD2 |
| ERBB3 | KMT2C |
| MDM2 | NOTCH2NL |
| TGFBR2 | KMT2C |
| FOXP2 | KMT2C |
| FOXP2 | TP53 |
| ATXN2 | BCOR |
| NOTCH2NL | FANCA |
| EP300 | BRCA2 |
| KMT2C | NOTCH2NL |
| KMT2C | NOTCH2NL |
| MAML2 | KMT2C |
| ADAM29 | NOTCH2 |
| GNAQ | KEAP1 |
| MLH1 | CYLD |
| NOTCH2NL | KMT2D |
| EOMES | ADAM29 |
| BCOR | ADAM29 |
| NOTCH2NL | KMT2D |
| FANCA | KMT2D |
| MEN1 | ADAM29 |
| NOTCH2NL | AR |
| NOTCH2 | ARID1B |
| BRCA2 | KMT2C |
| ERRFI1 | MDM2 |
| MSH2 | NOTCH2NL |
| MAML2 | KMT2D |
| CDKN2A | NF1 |
| TSC1 | KMT2C |
| TSC1 | RGPD3 |
| KMT2C | KMT2A |
| KMT2C | FAT1 |
| KMT2C | FLT1 |
| RGPD3 | KMT2C |
| SPTA1 | KMT2C |
| PALB2 | RGPD5 |
| FLT1 | NOTCH2 |
| KEAP1 | RGPD4 |
| MSH6 | RGPD3 |
| KMT2C | ATRX |
| FOXP2 | FANCD2 |
| NOTCH2 | KMT2C |
| FAT1 | TGFBR2 |
| BRAF | NOTCH2NL |
| KMT2C | KMT2D |
| PTEN | MSH2 |
| KMT2A | KMT2D |
| MSH2 | MSH2 |
| RPTOR | FAM135B |
| RUNX1 | RUNX1 |
| TCF7L2 | KMT2D |
| PDGFRA | KMT2D |
| NOTCH2NL | KMT2D |
| ADAM29 | EGFR |
| KMT2C |  |
| KMT2C |  |
| ARID1B |  |
| TP53 |  |
| ATXN1 |  |
| PKD2 |  |
