# Supplementary material for: Characterization of genetic alterations in brain metastases from non‐small cell lung cancer
Source: FEBS Open Bio. 2018 Aug 30;8(9):1544–52. doi: 10.1002/2211-5463.12501 (PMC6120240; doi:10.1002/2211-5463.12501)
Supplement: Supplementary file 5 — Table S5. Mutant genes identified in P5 primary tumor and matched brain metastasis samples. [file FEB4-8-1544-s005.docx]

**Supplemental table 5. Mutant genes identified in P5 primary tumors and matched brain metastases samples.**

| **P5- primary tumors** | **P5- brain metastases** |
| --- | --- |
| RUNX1 | ADAM29 |
| ADAM29 | RUNX1 |
| KMT2D | CREBBP |
| PIK3CB | PIK3CB |
| KMT2C | MSH2 |
| FANCD2 | STAG2 |
| KMT2C | TSC1 |
| ADAM29 | KMT2C |
| NOTCH2NL | KMT2C |
| KMT2C | TSC1 |
| GNAS | KMT2C |
| TP53 | PTK2 |
| AR | KMT2D |
| TNK2 | BARD1 |
| NOTCH2NL | MSH2 |
| FANCD2 | KMT2C |
| NOTCH2NL | KMT2C |
| ERRFI1 | NOTCH2NL |
| KMT2C | GNAS |
| KMT2C | TP53 |
| ADAM29 | AR |
| KMT2C | TNK2 |
| NOTCH2 | ERRFI1 |
| KMT2D | FANCD2 |
| ADAM29 | NOTCH2NL |
| ADAM29 | NOTCH2NL |
| ADAM29 | KMT2C |
| KMT2D | KMT2C |
| KMT2D | KMT2D |
| KMT2D | KMT2C |
| KMT2D | NOTCH2 |
| KMT2C | KMT2D |
| KMT2D | ADAM29 |
| NOTCH2NL | ADAM29 |
| KMT2D | ADAM29 |
| POLE | KMT2D |
| KMT2C | KMT2D |
| RGPD3 | KMT2D |
| ARID1B | KMT2D |
| KMT2C | KMT2D |
| KMT2C | KMT2D |
| NOTCH2 | KMT2C |
| FAS | KMT2D |
| KMT2C | KMT2D |
| NOTCH2NL | NOTCH2NL |
| KMT2D | POLE |
| KMT2D | KMT2D |
| ADAM29 | KMT2C |
| RUNX1 | KMT2D |
| KMT2D | KMT2D |
| KMT2D | KMT2D |
| KAT6A | KMT2D |
| EGFR | KMT2D |
|  | ARID1B |
|  | KMT2C |
|  | KMT2C |
|  | NOTCH2 |
|  | FAS |
|  | KMT2C |
|  | RGPD3 |
|  | NOTCH2NL |
|  | PTEN |
|  | KMT2D |
|  | KMT2D |
|  | MSH2 |
|  | KMT2D |
|  | PTEN |
|  | KMT2C |
|  | KMT2D |
|  | KMT2D |
|  | MAP4K5 |
|  | KMT2D |
|  | KAT6A |
|  | RUNX1 |
|  | KAT6A |
|  | EGFR |
