# Supplementary material for: Characterization of genetic alterations in brain metastases from non‐small cell lung cancer
Source: FEBS Open Bio. 2018 Aug 30;8(9):1544–52. doi: 10.1002/2211-5463.12501 (PMC6120240; doi:10.1002/2211-5463.12501)
Supplement: Supplementary file 6 — Table S6. Mutant genes identified in P6 primar tumors and matched brain metastasis samples. [file FEB4-8-1544-s006.docx]

**Supplemental table 6. Mutant genes identified in P6 primary tumors and matched brain metastases samples.**

| **P6- primary tumors** | **P6- brain metastases** |
| --- | --- |
| RUNX1 | RUNX1 |
| ADAM29 | ADAM29 |
| KRAS | KMT2D |
| NF2 | KRAS |
| INHBA | INHBA |
| LRP1B | LRP1B |
| KMT2C | TAF1L |
| TSC1 | FH |
| TSC1 | KMT2C |
| KMT2D | TSC1 |
| NOTCH2NL | KMT2C |
| KMT2C | PTCH1 |
| KMT2C | NOTCH2NL |
| PTCH1 | KMT2C |
| MYC | NSD1 |
| NSD1 | MYC |
| NOTCH2NL | NOTCH2NL |
| NOTCH2NL | NOTCH2NL |
| KMT2C | INPP4B |
| INPP4B | KMT2C |
| KMT2C | KMT2C |
| KMT2C | SLIT2 |
| KDM5A | NOTCH2 |
| SLIT2 | FLT4 |
| BCOR | NOTCH2 |
| EPHA2 | ADAM29 |
| NOTCH2 | ADAM29 |
| FLT4 | ADAM29 |
| CDKN2B | AR |
| KMT2D | KMT2D |
| ADAM29 | KMT2C |
| ADAM29 | NOTCH2NL |
| AR | KMT2D |
| KMT2D | FH |
| KMT2D | KMT2D |
| KMT2D | KMT2D |
| KMT2D | KMT2D |
| KMT2C | RGPD3 |
| FH | MET |
| KMT2D | KDR |
| NOTCH2NL | TNK2 |
| ADAM29 | KMT2C |
| KMT2D | KMT2C |
| KMT2D | NOTCH2 |
| KMT2D | INHBA |
| KMT2D | MSH2 |
| LRP1B | RGPD3 |
| RGPD3 | FANCD2 |
| MET | KMT2C |
| TNK2 | SKIDA1 |
| KDR | CREBBP |
| KMT2C | TBX3 |
| NOTCH2 | KMT2D |
| INHBA | ADAM29 |
| KMT2C | NOTCH2NL |
| RGPD3 | KMT2D |
| MSH2 | KMT2D |
| FANCD2 | ADAM29 |
| KMT2C | RUNX1 |
| TBX3 | TP53 |
| SKIDA1 | KMT2D |
| PTEN | NOTCH2NL |
| NOTCH2NL |  |
| NOTCH2NL |  |
| ADAM29 |  |
| KMT2D |  |
| KMT2D |  |
| KMT2C |  |
| KMT2D |  |
| RUNX1 |  |
| TP53 |  |
| KMT2D |  |
| KAT6A |  |
