# Supplementary material for: Characterization of genetic alterations in brain metastases from non‐small cell lung cancer
Source: FEBS Open Bio. 2018 Aug 30;8(9):1544–52. doi: 10.1002/2211-5463.12501 (PMC6120240; doi:10.1002/2211-5463.12501)
Supplement: Supplementary file 7 — Table S7. Shared pathogenic genes of six corresponding primary tumors and brain metastases (661 in total). [file FEB4-8-1544-s007.docx]

**Supplemental table 7: Shared pathogenic genes of 6 corresponding primary tumors and brain metastases (661 in total)**

| PDE4DIP | CNP | FRG1 | CHST3 | PCDHGA2 | FREM2 |
| --- | --- | --- | --- | --- | --- |
| HRNR | NCOR1 | TLR1 | GPRIN2 | GABRR2 | AKAP6 |
| NBPF10 | TBC1D3B | WFS1 | GSTO1 | RHAG | MMP14 |
| GPR137B | GPR142 | COMMD8 | COL17A1 | HIST1H1E | CHGA |
| OBSCN | PLXDC1 | TDO2 | NPY4R | GPR126 | PCK2 |
| NBPF15 | CDC27 | FAM160A1 | SLC16A9 | SYNE1 | ALDH6A1 |
| EVI5 | FBXO47 | APBB2 | NEBL | LAMA2 | RALGAPA1 |
| MYOC | TRIM16 | PARP8 | BBOX1 | CLPS | DYX1C1 |
| ITLN1 | ENO3 | FGFR4 | OR8B2 | FOXP4 | ADAMTS7 |
| NBPF8 | MARCH10 | CCDC125 | MAPK8IP1 | NCR2 | FAN1 |
| OR2T3 | INCA1 | GZMK | LRRC4C | AHI1 | VPS13C |
| VPS13D | USP6 | PCDHA4 | NEU3 | FBXO30 | OCA2 |
| RPE65 | ABCA10 | BCLAF1 | BAD | EGFR | SORD |
| MTOR | SPDYE4 | ENPP1 | ABCC8 | NFE2L3 | TJP1 |
| OR2T34 | KRT15 | NUP153 | OR4C12 | FBXO24 | SLC7A6 |
| TARBP1 | NFE2L1 | PACSIN1 | NCAPD3 | BBS9 | TNRC6A |
| MST1L | ABCA8 | PPP1R14C | TMEM25 | AEBP1 | PRR25 |
| PLEKHM2 | SPPL2C | DSP | NUP107 | GPR146 | SMPD3 |
| CASZ1 | LPIN2 | OOEP | OR6C4 | KMT2E | CACNA1H |
| TMEM51 | ZNF562 | SPACA1 | CD63 | COA1 | ZNF469 |
| EPHX1 | ZNF780B | DAAM2 | USP5 | LRGUK | PHLPP2 |
| PPIAL4G | PEG3 | TAAR5 | SLC15A5 | GNA12 | MSLN |
| PRAMEF4 | LPPR2 | NCOA7 | PHC1 | TTC26 | ADAMTS18 |
| VTCN1 | DUS3L | MRPL2 | KRT6B | PON3 | CNGB1 |
| EFNA4 | PLEKHA4 | TBC1D7 | ZMYM5 | NACAD | ESRP2 |
| HNRNPCL1 | TYK2 | UTRN | EXOSC8 | PABPC1 | MLYCD |
| PAM16 | KRI1 | ECI2 | RNF6 | ZNF16 | TMEM104 |
| PLA2G4A | ZNF181 | SLC22A23 | PABPC3 | NEFM | MYH2 |
| NOTCH2NL | RYR1 | DST | KLF5 | EYA1 | TEKT1 |
| MFSD2A | PLA2G4C | CTGF | CKAP2 | BAI1 | CCDC144NL |
| CALML6 | NWD1 | CDC40 | SIPA1L1 | MAPK15 | UTS2R |
| LAMC2 | NAPSA | TIAM2 | SEL1L | DENND3 | TXNDC2 |
| TAS1R1 | CCDC151 | GPR63 | RPGRIP1 | FAM83H | EMILIN2 |
| CSMD2 | RDH13 | HLA-DQB1 | FAM181A | CSMD1 | CEP192 |
| OXCT2 | VIT | PSPH | POTEG | MYOM2 | C18orf63 |
| RGS4 | CCT7 | PRSS1 | FANCI | INTS8 | SBK2 |
| MRPL24 | MYO7B | IRF5 | EIF2AK4 | LRRC6 | MFSD12 |
| ZSWIM5 | SLC8A1 | WIPI2 | ASB7 | CYSRT1 | CNN2 |
| DDAH1 | WNT10A | KMT2C | CATSPER2 | NFX1 | ZNF302 |
| PDC | MTX2 | DNAH11 | WDR72 | PPP1R26 | LILRA6 |
| EIF2B3 | TUBA3E | POM121C | MAN2C1 | ROR2 | ZNF317 |
| MTHFR | COL6A3 | C7orf25 | TYRO3 | IZUMO3 | C19orf35 |
| KDF1 | PARD3B | SEMA3D | CORO7 | MED27 | YJEFN3 |
| SEMA4G | ADNP | PLEC | CES1 | OR1L3 | PDE4A |
| TIMM23 | PROKR2 | NSMCE2 | GOT2 | FIBCD1 | ZNF486 |
| ZFYVE27 | MIR1-1HG | SDR16C5 | RRN3 | AFF2 | TIMM50 |
| NRAP | CRNKL1 | PCMTD1 | C16orf95 | STARD8 | PLIN4 |
| TACC2 | HCK | PLAT | ZFPM1 | SLC38A5 | FBL |
| ADAMTS14 | TLDC2 | GPT | E4F1 | GAB3 | SDHAF1 |
| LRRC18 | LZTS3 | AK8 | RPUSD1 | RPGR | ZNF320 |
| ABI1 | ZMYND8 | VPS13A | TMEM204 | HIVEP3 | ZNF565 |
| IDE | AP5S1 | FANCC | ABR | NAV1 | ZNF708 |
| PNLIP | URB1 | AQP7 | TP53 | PRDM16 | NOSTRIN |
| EIF5AL1 | PFKL | NOL8 | DNAH9 | KIAA1324 | TTN |
| UPK2 | TPTE | PCSK5 | OR1E1 | PLEKHG5 | CYP26B1 |
| TRPM5 | C21orf58 | CCBL1 | EPN3 | SHC1 | POTEE |
| SAA2 | UPB1 | CACNA1B | USP32 | EPHA2 | HSPD1 |
| OR9G1 | SERPIND1 | PRSS3 | SPATA20 | CR1L | CHAC2 |
| OR10G4 | PIM3 | ABCA1 | ENTHD2 | LGR6 | SLC16A14 |
| MUC5B | OSBP2 | NDOR1 | SLFN14 | DRAXIN | SMYD1 |
| MUC6 | TRMU | OR1Q1 | ZADH2 | C1orf64 | CCDC74B |
| NLRP14 | LGALS2 | OR13C5 | POTEC | INPP5B | ITPRIPL1 |
| LRP4 | TTLL12 | SLC25A5 | TICAM1 | PYGO2 | EDAR |
| HPS5 | SUSD2 | ARSD | SPHK2 | PRPF38A | MYT1 |
| ROBO4 | ACKR4 | BCOR | RSPH6A | PYCR2 | TUBB1 |
| ESRRA | NKTR | RBMX | MUC16 | ZMYM6 | TMEM74B |
| MAP4K2 | LSMEM2 | GPR112 | BBC3 | CACNA1S | SLC17A9 |
| OR10G7 | POMGNT2 | EGFL6 | ZNF208 | LAD1 | APCDD1L |
| OR4C3 | PTPN23 | CDK16 | SPTBN4 | ATP6V1G3 | TMPRSS3 |
| OR8U1/8 | PLXND1 | ARSE | FCGBP | PADI4 | PRDM15 |
| SAA1 | IQSEC1 | MAGI1 | DMRTC2 | S100PBP | DEPDC5 |
| TAS2R19 | SIDT1 | WASF2 | OR7E24 | ATAD3B | ZNF70 |
| TCP11L2 | MUC20 | PDE4DIP | OR7A5 | CELSR2 | KIAA1143 |
| TAS2R31 | FLNB | HSPA6 | ZNF14 | TNN | MCM2 |
| KCNC2 | P4HTM | POGK | AKAP8L | TESK2 | P2RY14 |
| TAS2R46 | TMEM43 | BRDT | LTBP1 | OR2T27 | CSRNP1 |
| C1RL | C3orf30 | CD2 | XIRP2 | SVIL | MED12L |
| TAS2R43 | CACNA1D | SMYD3 | GPR1 | FBXW4 | SFMBT1 |
| KLRC2 | SLC22A13 | WDR64 | ANKRD36 | WDFY4 | CD80 |
| TAS2R30 | ZNF717 | EPS8L3 | EML6 | RRP12 | C3orf17 |
| TRMT112 | DYSF | OR4D5 | BAZ2B | CDHR1 | DMBT1 |
| ZNHIT2 | PXDN | OR51A4 | MTIF2 | RTKN2 | OR8D4 |
| BMS1 | FIBIN | NPBWR2 | COMTD1 | TRIM8 | OR8U1 |
| NPBWR1 | ANK2 | KRT18 | PPAP2B | ZFHX3 | EFCAB14 |
| EPPK1 | HERC5 | ALDH1L2 | TOMM40L | MAP2K3 | ZNF239 |
| PEBP4 | RNF212 | IRAK4 | KIAA1522 | KCNJ12 | SYT15 |
| GINS4 | ZNF141 | CEP290 | EPB41 | CPD | FAM160B1 |
| C8orf58 | UVSSA | PIBF1 | GPATCH2 | LRRC37A3 | BICC1 |
| C9orf135 | HMGCR | PARP4 | DENND1B | CNTROB | RBM20 |
| SURF6 | PCDHA6 | BIVM | TNR | MYO18A | RET |
| ENTPD2 | SLC6A7 | STARD13 | NIT1 | CLSTN2 | ECHDC3 |
| NUTM2G | ARHGAP25 | WDR89 | LEFTY1 | EPHA3 | PRR32 |
| FOXD4L5 | USP37 | OXA1L | SLC44A3 | NEK4 | DDX53 |
| RASEF | REG3A | OR4K2 | ITLN2 | TPRA1 | OR51G2 |
| TNMD | NINL | MAP4K5 | KIF2C | WWC2 | MRGPRX3 |
| GPC4 | RPRD1B | EXOC3L4 | AP4B1 | HTT | ARHGAP32 |
| MID1 | BPIFB4 | NRDE2 | FOXJ3 | MRPL1 | OR51Q1 |
| GLA | ATP9A | ANKRD34C | MAGI3 | BST1 | TH |
| TAZ | CNBD2 | OR4M2 | ASAP3 | SH3TC1 | ST5 |
| MACROD2 | ADIG | ZSCAN2 | TMEM52 | ACSL1 | OR4D6 |
| PRDM6 | PRPF40B | PHGR1 | FH | CRMP1 | MOGAT2 |
| SPEF2 | KERA | OR4N4 | RGS16 | ZNF732 | PTPRB |
| TTC33 | PAH | IFT140 | MAST2 | SRP72 | SLC38A4 |
| MRPS27 | KMT2D | SPIRE2 | CSF3R | MAML3 | OR6C3 |
| GPR98 | KRT73 | ACSM2B | ASPM | ERBB2IP | EP400 |
| PAM | NUDT15 | PKHD1 | SLC2A10 | ZFAND2A | CLTCL1 |
| HSD17B4 | IFT88 | ITPR3 | BPIFB3 | SCRN1 | KIAA0930 |
| CROT | CPT1B | TFEB | TRPC4AP | SSBP1 | ISX |
| SAMD9L | TCN2 | HMGCLL1 | KRTAP10-2 | C7orf50 | COLQ |
| RP1L1 | SNRK | HLA-A | EP300 | ZNF138 | KIF9 |
| SPAG11B | |  |  |  |  |
