# Supplementary material for: Characterization of genetic alterations in brain metastases from non‐small cell lung cancer
Source: FEBS Open Bio. 2018 Aug 30;8(9):1544–52. doi: 10.1002/2211-5463.12501 (PMC6120240; doi:10.1002/2211-5463.12501)
Supplement: Supplementary file 8 — Table S8. The gene ontology biological processes (GO‐BP) analysis of shared pathogenic genes of corresponding primary tumors and metastases. [file FEB4-8-1544-s008.docx]

**Supplemental table 8. The gene ontology biological processes (GO-BP) analysis of shared pathogenic genes of corresponding primary tumors and metastases**

| **Term** | **Gene ontology biological processes (GO-BP)** | **P-Value** |
| --- | --- | --- |
| GO:0007186 | G-protein coupled receptor signaling pathway | 1.82E-05 |
| GO:0015908 | fatty acid transport | 0.006873268 |
| GO:0031581 | hemidesmosome assembly | 0.006873268 |
| GO:0034968 | histone lysine methylation | 0.01081065 |
| GO:0045053 | protein retention in Golgi apparatus | 0.010817152 |
| GO:0007076 | mitotic chromosome condensation | 0.013176412 |
| GO:0018108 | peptidyl-tyrosine phosphorylation | 0.015710811 |
| GO:0035855 | megakaryocyte development | 0.015813557 |
| GO:0051056 | small GTPase mediated signal transduction | 0.016377494 |
| GO:0006623 | protein targeting to vacuole | 0.028300307 |
| GO:0046902 | mitochondrial membrane permeability | 0.043484938 |
