# Supplementary material for: Characterization of genetic alterations in brain metastases from non‐small cell lung cancer
Source: FEBS Open Bio. 2018 Aug 30;8(9):1544–52. doi: 10.1002/2211-5463.12501 (PMC6120240; doi:10.1002/2211-5463.12501)
Supplement: Supplementary file 9 — Table S9. The gene ontology cellular component (GO‐CC) analysis of shared pathogenic genes of corresponding primary tumors and metastases. [file FEB4-8-1544-s009.docx]

**Supplemental table 9. The gene ontology cellular component (GO-CC) analysis of shared pathogenic genes of corresponding primary tumors and metastases**

| **Term** | **Gene ontology cellular component (GO-CC)** | **P-Value** |
| --- | --- | --- |
| GO:0016605 | PML body | 0.046883216 |
| GO:0005796 | Golgi lumen | 0.042718869 |
| GO:0005578 | proteinaceous extracellular matrix | 0.03786386 |
| GO:0005929 | cilium | 0.03133346 |
| GO:0005856 | cytoskeleton | 0.026295869 |
| GO:0005887 | integral component of plasma membrane | 0.026007087 |
| GO:0005938 | cell cortex | 0.022899014 |
| GO:0005782 | peroxisomal matrix | 0.018524522 |
| GO:0005886 | plasma membrane | 0.009919439 |
| GO:0042383 | sarcolemma | 0.007696964 |
| GO:0019898 | extrinsic component of membrane | 0.006218198 |
| GO:0030056 | hemidesmosome | 0.001859995 |
