# Supplementary material for: Characterization of genetic alterations in brain metastases from non‐small cell lung cancer
Source: FEBS Open Bio. 2018 Aug 30;8(9):1544–52. doi: 10.1002/2211-5463.12501 (PMC6120240; doi:10.1002/2211-5463.12501)
Supplement: Supplementary file 10 — Table S10. The gene ontology molecular function (GO‐MF) analysis of shared pathogenic genes of corresponding primary tumors and metastases. [file FEB4-8-1544-s010.docx]

**Supplemental table 10. The gene ontology** molecular function **(GO-MF) analysis of shared pathogenic genes of corresponding primary tumors and metastases**

| **Term** | **Gene ontology molecular function (GO-MF)** | **P-Value** |
| --- | --- | --- |
| GO:0004930 | G-protein coupled receptor activity | 2.37E-05 |
| GO:0030506 | ankyrin binding | 4.60E-04 |
| GO:0018024 | histonelysine N-methyltransferase activity | 0.001922 |
| GO:0004888 | transmembrane signaling receptor activity | 0.002811 |
| GO:0005096 | GTPase activator activity | 0.030091 |
| GO:0005200 | structural constituent of cytoskeleton | 0.034564 |
| GO:0035257 | nuclear hormone receptor binding | 0.037582 |
| GO:0004713 | protein tyrosine kinase activity | 0.03858 |
| GO:0004714 | transmembrane receptor tyrosine kinase activity | 0.039633 |
| GO:0016746 | transferase activity | 0.043015 |
| GO:0005245 | voltage-gated calcium channel activity | 0.046555 |
