# Supplementary material for: Characterization of genetic alterations in brain metastases from non‐small cell lung cancer
Source: FEBS Open Bio. 2018 Aug 30;8(9):1544–52. doi: 10.1002/2211-5463.12501 (PMC6120240; doi:10.1002/2211-5463.12501)
Supplement: Supplementary file 11 — Table S11. KEGG pathway analysis: 52 pathways associated with cancer‐related signaling. [file FEB4-8-1544-s011.docx]

**Supplemental table 11. KEGG pathway analysis:** 5**2 pathways associated with cancer-related signaling were displayed in below**

| **KEGG pathways** | **Count** | **Genes** |
| --- | --- | --- |
| Metabolic pathways | 31 | HSD17B4 PAH PFKL ACSL1 UPB1 CCBL1 ATP6V1G3 PCK2 SMPD3 PYCR2 ENPP1 MTHFR TH INPP5B PLA2G4A PPAP2B BST1 MLYCD ENO3 GOT2 GPT PON3 PSPH SPHK2 ALDH6A1 HMGCR TDO2 PNLIP CES1 SORD ACSM2B |
| MAPK signaling pathway | 13 | MAPK8IP1 FLNB CACNA1H CACNA1S CACNA1B CACNA1D MAP4K2 GNA12 MAP2K3 PLA2G4A FGFR4 HSPA6 EGFR |
| Calcium signaling pathway | 11 | SLC8A1 ITPR3 CACNA1H RYR1 CACNA1S CACNA1B CACNA1D SLC25A5 SPHK2 CALML6 EGFR |
| Focal adhesion | 9 | COL6A3 FLNB LAMC2 BAD SHC1 TNN LAMA2 TNR EGFR |
| Pathways in cancer | 9 | RET MTOR LAMC2 WNT10A BAD CSF3R EP300 LAMA2 EGFR |
| GnRH signaling pathway | 8 | ITPR3 MMP14 CACNA1S CACNA1D MAP2K3 PLA2G4A CALML6 EGFR |
| Fc gamma R-mediated phagocytosis | 6 | WASF2 PLA2G4A PPAP2B ASAP3 SPHK2 HCK |
| Toll-like receptor signaling pathway | 6 | MAP2K3 IRAK4 TICAM1 TLR1 CD80 IRF5 |
| Spliceosome | 6 | PRPF38A CRNKL1 RBMX PRPF40B HSPA6 CDC40 |
| RNA transport | 6 | NUP153 EIF2B3 PABPC3 POM121C PABPC1 NUP107 |
| RNA degradation | 5 | HSPD1 PABPC3 ENO3 PABPC1 EXOSC8 |
| Adherens junction | 5 | WASF2 PTPRB TJP1 EP300 EGFR |
| Peroxisome | 5 | HSD17B4 ACSL1 ECI2 MLYCD CROT |
| ECM-receptor interaction | 5 | COL6A3 LAMC2 TNN LAMA2 TNR |
| Gap junction | 5 | ITPR3 TUBB1 TUBA3E TJP1 EGFR |
| ABC transporters | 4 | ABCA1 ABCC8 ABCA8 ABCA10 |
| Glioma | 4 | MTOR SHC1 CALML6 EGFR |
| PPAR signaling pathway | 4 | CPT1B ACSL1 PCK2 AQP7 |
| Protein digestion and absorption | 4 | SLC8A1 COL6A3 PRSS3 COL17A1 |
| ErbB signaling pathway | 4 | MTOR BAD SHC1 EGFR |
| Prostate cancer | 4 | MTOR BAD EP300 EGFR |
| Cell cycle | 4 | CDC27 MCM2 MYT1 EP300 |
| Tight junction | 4 | MAGI3 MYH2 EPB41 TJP1 |
| Natural killer cell mediated cytotoxicity | 4 | NCR2 KLRC2 SHC1 HLA-A |
| Phagosome | 4 | ATP6V1G3 TUBB1 TUBA3E HLA-A |
| Glycolysis / Gluconeogenesis | 3 | PFKL PCK2 ENO3 |
| VEGF signaling pathway | 3 | PLA2G4A BAD SPHK2 |
| Antigen processing and presentation | 3 | KLRC2 HLA-A HSPA6 |
| Phosphatidylinositol signaling system | 3 | ITPR3 INPP5B CALML6 |
| Ribosome biogenesis in eukaryotes | 3 | BMS1 NRAP FBL |
| TGF-beta signaling pathway | 3 | LEFTY1 LTBP1 EP300 |
| Melanogenesis | 3 | WNT10A EP300 CALML6 |
| Cell adhesion molecules (CAMs) | 3 | CD2 HLA-A CD80 |
| Wnt signaling pathway | 3 | DAAM2 WNT10A EP300 |
| Jak-STAT signaling pathway | 3 | TYK2 CSF3R EP300 |
| Chemokine signaling pathway | 3 | TIAM2 SHC1 HCK |
| Cytokine-cytokine receptor interaction | 3 | EDAR CSF3R EGFR |
| Protein export | 2 | SRP72 OXA1L |
| Base excision repair | 2 | MID1 PARP4 |
| DNA replication | 2 | MCM2 SSBP1 |
| Notch signaling pathway | 2 | MAML3 EP300 |
| Endometrial cancer | 2 | BAD EGFR |
| Non-small cell lung cancer | 2 | BAD EGFR |
| Acute myeloid leukemia | 2 | MTOR BAD |
| p53 signaling pathway | 2 | BBC3 BAI1 |
| Pancreatic cancer | 2 | BAD EGFR |
| Melanoma | 2 | BAD EGFR |
| Chronic myeloid leukemia | 2 | BAD SHC1 |
| Fc epsilon RI signaling pathway | 2 | MAP2K3 PLA2G4A |
| mRNA surveillance pathway | 2 | PABPC3 PABPC1 |
| Small cell lung cancer | 2 | LAMC2 LAMA2 |
| Apoptosis | 2 | IRAK4 BAD |
